# Time Course Transcriptome Analysis of Spina Bifida Progression in Fetal Rats

**DOI:** 10.3390/brainsci11121593

**Published:** 2021-11-30

**Authors:** Kendall P. Murphy, Bedika Pathak, Jose L. Peiro, Marc Oria

**Affiliations:** 1 Department of Orthopaedic Surgery, University of Cincinnati, Cincinnati, OH 45267, USA; murphk9@ucmail.uc.edu; 2 Center for Fetal and Placental Research, Division of Pediatric General and Thoracic Surgery, Cincinnati Children’s Hospital and Medical Center, Cincinnati, OH 45229, USA; pathakba@mail.uc.edu (B.P.); jose.peiro@cchmc.org (J.L.P.); 3 Department of Pediatrics, University of Cincinnati, Cincinnati, OH 45267, USA

**Keywords:** spina bifida, transcriptomics, RNA sequencing, neural tube defects

## Abstract

A better understanding of the transcriptomic modifications that occur in spina bifida may lead to identify mechanisms involved in the progression of spina bifida in utero and the development of new therapeutic strategies that aid in spinal cord regeneration after surgical interventions. In this study, RNA-sequencing was used to identify differentially expressed genes in fetal spinal cords from rats with retinoic acid-induced spina bifida at E15, E17, and E20. Gene ontology, KEGG, and protein–protein interaction analysis were conducted to predict pathways involved in the evolution of the disease. Approximately 3000, 1000 and 300 genes were differentially expressed compared to the control groups at E15, E17 and E20, respectively. Overall, the results suggest common alterations in certain pathways between gestational time points, such as upregulation in p53 and sonic hedgehog signaling at E15 and E17 and downregulation in the myelin sheath at E17 and E20. However, there were other modifications specific to gestational time points, including skeletal muscle development at E15, downregulated glucose metabolism at E17, and upregulated inflammation at E20. In conclusion, this work provides evidence that gestational age during spina bifida repair may be a significant variable to consider during the development of new regenerative therapeutics approaches.

## 1. Introduction

Myelomeningocele (MMC), the most significant form of spina bifida, is a devastating congenital malformation of the spinal cord associated with severe morbidity and mortality [1]. This “two-hit” process occurs during the folding of the neural plate into the neural tube during early development, 3–4 weeks of gestation, resulting in the lack of sclerotomal coverage leaving the neural tissue directly exposed to the amniotic fluid [2,3], called the “first hit” followed by an in utero acquired neurodegeneration by the chemical action of the amniotic fluid to the neural tissue or “second hit”. This can result in severe consequences, including decreased mobility and limb paralysis, bladder and intestinal incontinence, and stunted neurological function [4]. Evidence indicates that consequences progress in severity if not corrected in utero; however, fetal repair only stops progression in most cases, as it does not reverse the existing damage [5,6,7].

Despite the advances in surgical techniques for spina bifida repair in utero, effective regenerative treatments for the devastating neurotological alteration have not yet been developed. To develop better therapeutic approaches, it is extremely important to understand the molecular changes present in the neural tissue once the defect has occurred and during this progressive degeneration in utero. The analysis of transcriptome studies at different time points throughout gestation would provide in-depth knowledge of the regulatory changes present in the neural tissue of the neurodegenerative progression in utero after spina bifida occurs. Through these studies, pathways could be identified as therapeutic targets to aid in spinal cord regeneration.

In a follow-up study to our previous publication [8], we have performed a comprehensive time-course transcriptomic analysis of the commonly used congenital retinoic acid (RA)-induced spina bifida rat model to characterize the progressive changes that occur in the neural tissue after the development of spina bifida [4]. Gene expression was analyzed in the spinal cords of three fetal gestational points: E15, E17, and E20 of fetuses with spina bifida and controls, using RNA sequencing. The justification of these time points was based on lung and spinal cord development since lung development is known as an indicator of maturity and organogenesis when comparing humans and rodents. Lung development predominantly occurs during the canalicular–saccular phase in mice from E17 to birth. Comparatively, this reflects changes that occur during weeks 15–38 of gestation in humans [9]. Additionally, spinal cord neurogenesis begins at E11 and peaks around E17 in rodents with the beginning of the gliogenesis processes [10,11]. Furthermore, this reflects changes that occur during weeks 22–24 of gestation in humans. Therefore, these time points make sense in order to study the progression of the defect in response to amniotic fluid exposure. Additionally, we identify an important role of specific pathways along with the progression of the disease in utero. We conclude that these results will guide future studies of strategies to regenerate spinal cord tissue after or during in utero spina bifida repair [12].

## 2. Materials and Methods

### 2.1. Animals

Sprague Dawley rats weighing 200–250 g (Charles River Laboratories, Inc., Wilmington, NC, USA) were housed at 22 °C in a standard dark:light cycle (10:14 h) with access to water and standard food ad libitum. The mating date was defined as E-1 and plug day as E0. The experimental protocol was in agreement with the National Institutes of Health Guidelines for Care and Use of Laboratory Animals and was approved by the Institutional Animal Care and Use Committee at Cincinnati Children’s Hospital and Medical Center (IACUC 2019-0081).

### 2.2. Congenital Retinoic Acid (RA) Induced Spina Bifida Animal Model

On E10 at 10:00 a.m., pregnant dams were gavaged with 100 mg/kg trans-retinoic acid (RA) (Sigma Aldrich, St. Louis, MO, USA) solubilized in olive oil or an equal volume of olive oil only (vehicle). With this model, 70% of fetuses in each liter were diagnosed with spina bifida based on open spinal defect [8]. At E15, E17, and E20, pregnant dames were humanely sacrificed and fetuses from three different groups were harvested: (i) MMC—open spinal cords from fetuses with RA-induced spina bifida; (ii) control—spinal cords from non-affected siblings from mothers who received Ra; and (iii) vehicle—spinal cords from fetuses whose mothers received olive oil only.

### 2.3. Tissue Processing and RNA Extraction

At E15, E17, and E20, comparable size spinal cords from vehicle, control, and MMC fetuses were dissected from the lumbar region and snap-frozen and stored at −80 °C until used for gene expression analysis. In MMC fetuses, only the spinal cord from the open area were dissected. Frozen spinal cords were homogenized using an IkaT10 basic Ultra-Turrax homogenizer in RLT buffer and then RNA was extruded using the RNeasy Plus Mini Kit (Qiagen Science, Hilden, Germany) following the manufacturer’s protocol. RNA quantity was assessed through spectrophotometric analysis using an Epoch Biotek spectrophotometer (Biotek Instruments, Winooski, VT, USA).

### 2.4. cDNA Library Preparation and Sequencing

Agarose gels (1%) were used to monitor RNA degradation and contamination. RNA purity was assessed using the NanoPhotometer^®^ spectrophotometer (Implen, West Lake Village, CA, USA). RNA concentration was measured using the Qubit^®^ RNA Assay Kit in a Qubit^®^ 2.0 Fluorometer (Life Technologies, Carlsbad, CA, USA) and the RNA integrity was evaluated using the RNA Nano6000 Assay Kit for the Bioanalyzer 2100 system (Agilent Technologies, Santa Clara, CA, USA).

Three micrograms of total RNA per sample were used to generate sequencing libraries (*n* = 2 animals per group per time point). Libraries were generated using the NEBNext^®^ UltraTM RNA Library Prep Kit for Illumina^®^ (NEB, Ipswich, MA, USA) following the manufacturer’s recommendations. cDNA products were purified using the AMPure XP system and library quality was determined using the Agilent Bioanalyzer 2100 system. The cBot Cluster Generation System using the HiSeq PE Cluster Kit cBot-HS (Illumina, San Diego, CA, USA) was used to cluster the samples according to the manufacturer’s instructions. After this, the library products were sequenced on an Illumina HiSeq (Illumina, San Diego, CA, USA) and 125 bp/150 bp paired-end reads were generated per sample. 

### 2.5. RNA-Seq Data Processing and Analysis

Raw FastQ files were processed through in-house Perl scripts, where clean reads were obtained by removing reads containing adapter or poly(N) as well as low-quality reads. TopHat v2.0.12 was used to align clean reads to the reference genome, which was built using Bowtie v2.2.3. HTSeq v 0.6.1 was used to calculate the fragments per kilobase of exon per million base pairs mapped (FPKM) based on the gene’s length and reads mapped to that gene. Differential expression analysis was performed using the DESeqR software v1.18.0. The resulting *p* values less than 0.05 were considered differentially expressed. This analysis identified differentially expressed genes (DEGs) between control, vehicle, and MMC groups at E15, E17, and E20. Principal component analysis was conducted using the AltAnalyzer package v2.0 (http://www.altanalyze.org, accessed on 18 February 2021) [13,14,15]. The GOseqR package was used to perform gene ontology (GO) enrichment analysis and GO terms with a *p* value less than 0.05 were considered statistically significant. RNAseq data were deposited in the Sequence Read Archive (SRA) and can be found via BioProject (PRJNA683230) and SRA(PRJNA683793). The protein–protein interaction network (PPI) was constructed and illustrated using the search tool for the retrieval of interacting genes/proteins (STRING) (https://string-db.org/, accessed on 27 May 2021) database to reveal the relationships of the top 25 DEGs based on a minimum required interaction.

### 2.6. RT-qPCR Analysis

Utilizing the RT^2^ First Strand Kit (Qiagen, Germantown, MD, USA), 1 µg RNA /sample was reverse transcribed into cDNA. Four to six samples from the MMC and vehicle groups were analyzed. A 1 µg cDNA sample was then used as a template for RT-qPCR employing TaqMan^®^ gene expression assays (Applied Biosystems, Foster City, CA, USA) (Table 1) in the 7500 Fast Real-Time PCR system. Samples were run in duplicate for target genes and were normalized using HPRT1 as an endogenous control.

### 2.7. Statistical Analysis

All graphs were performed in GraphPad Prism9 software (GraphPad Software Inc., La Jolla, CA, USA). A fold change > 1.5 and *p* value < 0.05 was considered statistically significant. Relative quantification of transcript expression from RT-qPCR was performed using the 2^−ΔΔCt^ method comparing MMC and vehicle, where Ct represents the threshold cycle. Error bars indicate the standard error of the mean.

## 3. Results

### 3.1. Differentially Expressed Genes in the Fetal Spinal Cord after MMC Occurs

**Figure 1:**
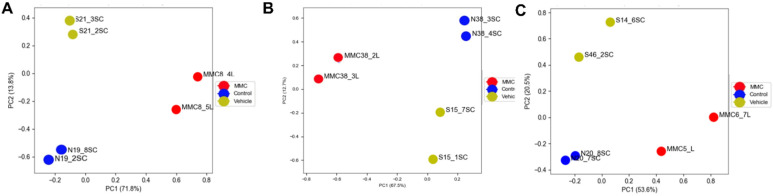
Principal component analysis of libraries sequenced for RNA-seq for tissues isolated at (**A**) E15, (**B**) E17, and (**C**) E20.

**Figure 2:**
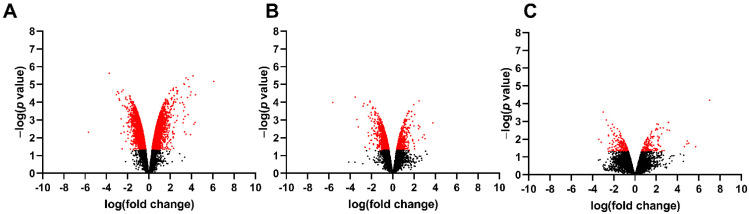
Volcano plot illustrating RNA-seq data of fetal spinal cords isolated from MMC and vehicle at (**A**) E15, (**B**) E17, and (**C**) E20. Red identifies genes that were greater than 1.5-fold change and *p* < 0.05.

**Figure 3:**
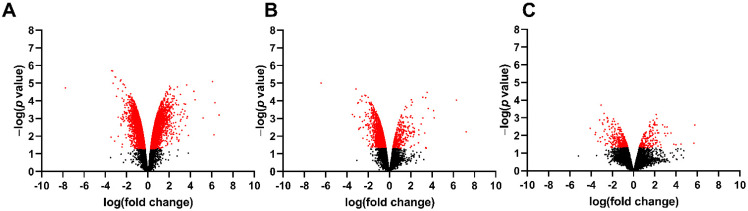
Volcano plot illustrating RNA-seq data of fetal spinal cords isolated from MMC and control at (**A**) E15, (**B**) E17, and (**C**) E20. Red identifies genes that were greater than 1.5-fold change and *p* < 0.05.

**Figure 4:**
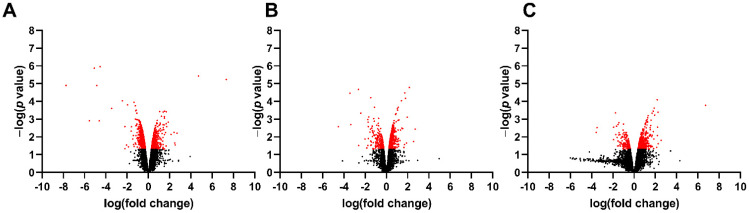
Volcano plot illustrating RNA-seq data of fetal spinal cords isolated from control and vehicle at (**A**) E15, (**B**) E17, and (**C**) E20. Red identifies genes that were greater than 1.5-fold change and *p* < 0.05.

To identify spinal cord changes in gene expression after MMC occurs in rat fetuses, we compared RNA-sequencing data between MMC, control, and vehicle groups at E15, E17, and E20. Principal component analysis indicates segregation of transcriptomes from the MMC group compared to the vehicle and control groups at each time point (Figure 1). The number of DEGs, identified as >1.5-fold change and *p* < 0.05 for each comparison at each time point is found in Table 2. Interestingly, the number of DEGs between MMC and either control or vehicle were greater than the control, compared to the vehicle, and the number of DEGs decreased between each comparison as gestation progressed. These trends are illustrated as volcano plots found in Figure 2 (MMC vs. vehicle), Figure 3 (MMC vs. control), and Figure 4 (control vs. vehicle).

**Figure 5:**
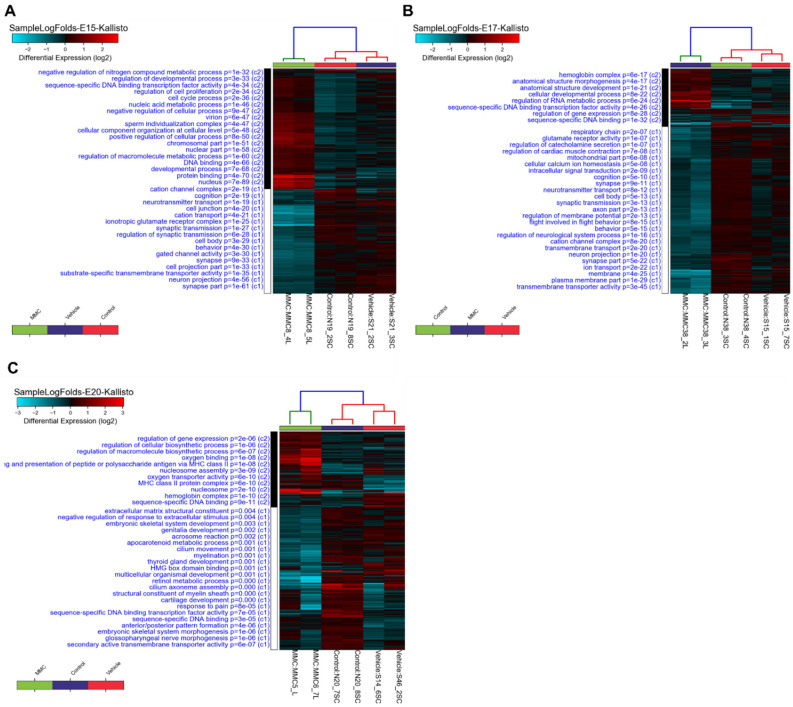
Hierarchical clustering analysis of differentially expressed genes in the spinal cord of MMC, vehicle, and control fetuses isolated at (**A**) E15, (**B**) E17, and (**C**) E20.

Hierarchical clustering analysis indicates that vehicle and control groups cluster together compared to the MMC group at each time point studied (Figure 5). These data indicate that at E15 and E17, genes involved in neurological systems function, such as synaptic transmission, synapse, neuron projection, and neurotransmitter transport, are downregulated in MMC compared to either control or vehicle (Figure 5A,B). Additionally, at E20, inflammatory genes, such as those involved in MHC class II antigen presentation, are upregulated, and genes involved in the development of the myelin sheath are downregulated in MMC compared to the control groups (Figure 5C). Because of this clustering pattern, we chose to focus our further analyses on the comparison between MMC and vehicle at each time point; however, the comparisons between MMC and control and control compared to the vehicle can be found in the Supplemental Information.

The 25 genes that are most significantly upregulated and downregulated between MMC and vehicle groups at E15, E17 and E20 are listed in Table 3, Table 4 and Table 5 respectively. Additionally, the top 25 upregulated and downregulated DEGs between MMC and control at E15, E17, and E20 are listed in Supplementary Tables S1–S3 respectively. Furthermore, the top 25 upregulated and downregulated DEGs between control and vehicle at each time point are listed in Supplementary Tables S4–S6.

Collectively, these results provide initial evidence that downregulated genes at E15 and E17 may lead to a decline in neural function and upregulated genes at E20 result in inflammation and disruption in myelin sheath development in rat fetuses with RA-induced MMC. 

### 3.2. GO Analysis of Differentially Expressed Genes

**Figure 6:**
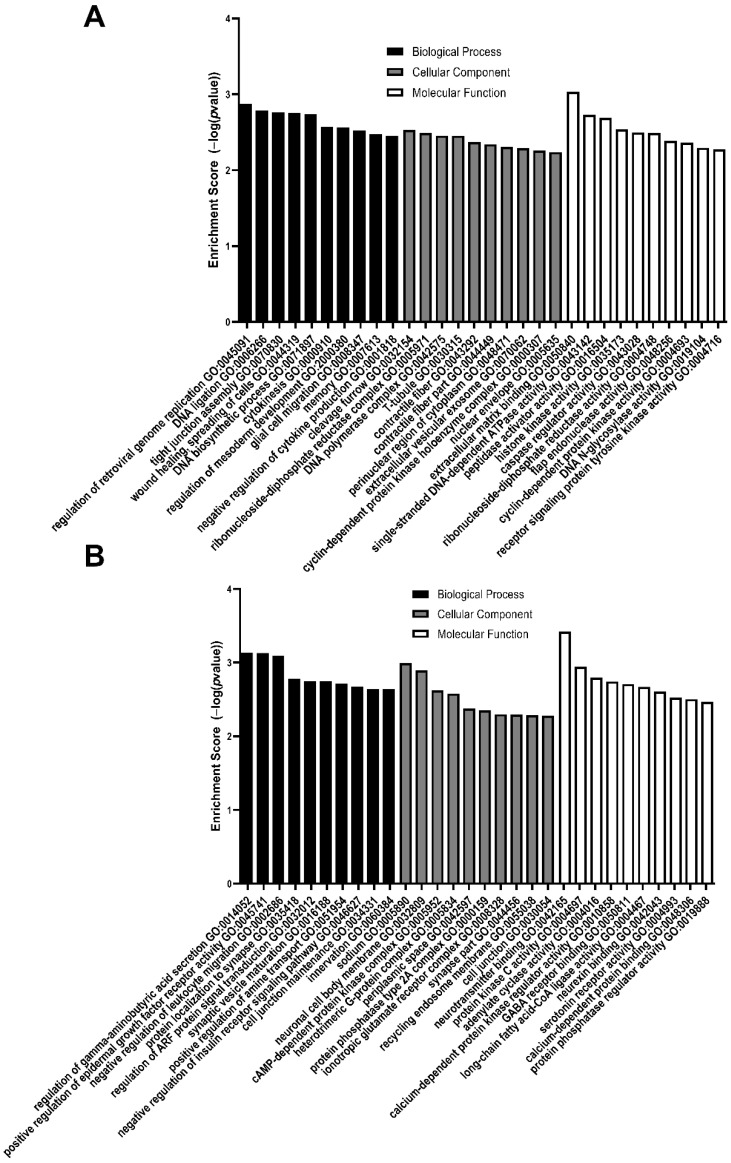
Enrichment scores of the top 10 GO biological processes, cellular components, and molecular function enriched from differentially expressed genes that are (**A**) upregulated or (**B**) downregulated in MMC spinal cords compared to vehicle at E15. All GO pathways were >1.5-fold and *p* < 0.05.

**Figure 7:**
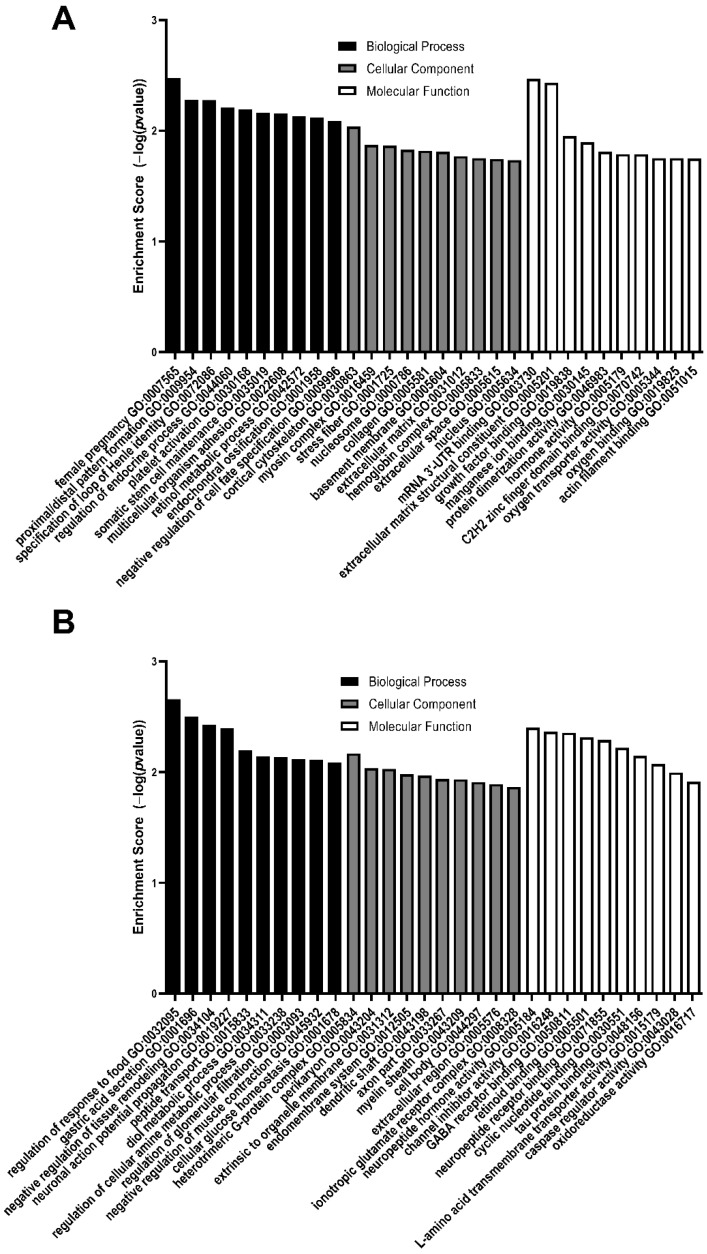
Enrichment scores of the top 10 GO biological processes, cellular components, and molecular function enriched from differentially expressed genes that are (**A**) upregulated or (**B**) downregulated in MMC spinal cords compared to vehicle at E17. All GO pathways were >1.5-fold and *p* < 0.05.

**Figure 8:**
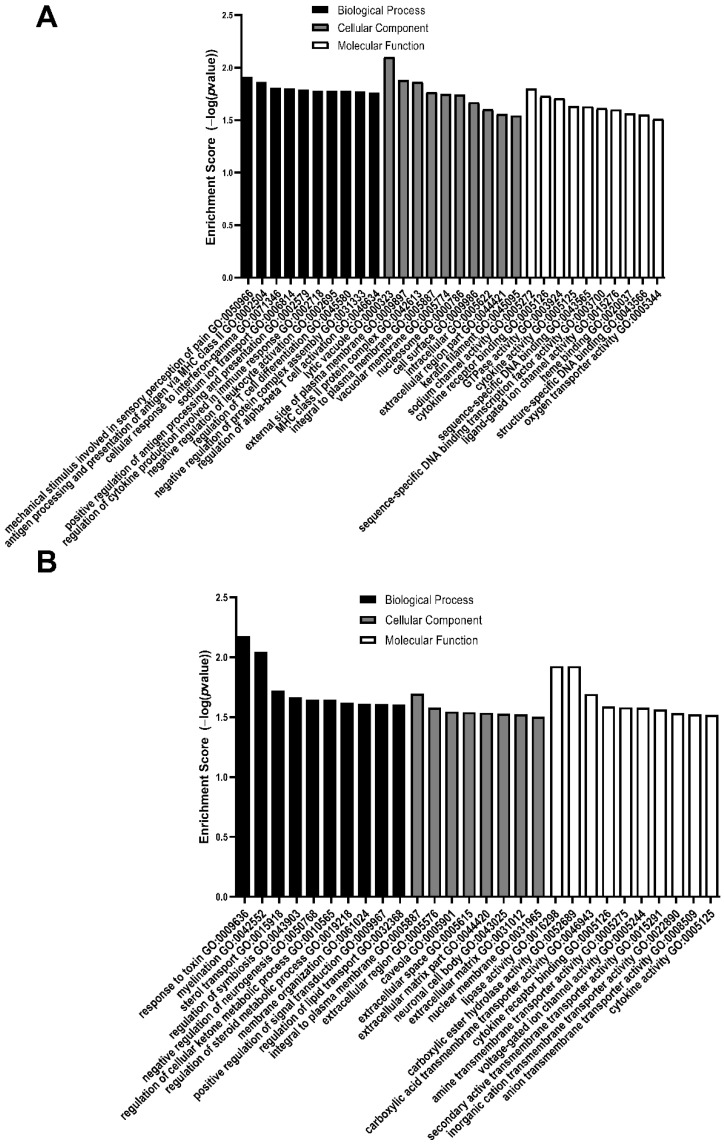
Enrichment scores of the top 10 GO biological processes, cellular components, and molecular function enriched from differentially expressed genes that are (**A**) upregulated or (**B**) downregulated in MMC spinal cords compared to vehicle at E20. All GO pathways were >1.5-fold and *p* < 0.05.

The top 10 enriched GO biological processes, cellular components, and molecular functions from upregulated and downregulated DEGs between MMC and vehicle at E15 are listed in Table 6 and Table 7, E17 in Table 8 and Table 9, and E20 in Table 10 and Table 11. Additionally, the enrichment score of each GO listed between MMC and vehicle at E15, E17, and E20 is depicted in Figure 6, Figure 7 and Figure 8, respectively. At E15, upregulated DEGs were enriched in biological processes, such as glial cell migration and regulation of mesoderm development, cellular components, such as contractile fiber and T-tubule, and molecular functions, such as extracellular matrix binding and caspase regulator activity (Table 6, Figure 6A). At E15, downregulated DEGs were enriched in biological processes, such as protein localization to synapse and synaptic vesicle maturation, cellular components, such as cell junction and ionotropic glutamate receptor complex, and molecular functions, such as calcium-dependent protein binding and GABA receptor binding (Table 7, Figure 6B).

At E17, upregulated DEGs were enriched in biological processes, including proximal/distal pattern formation and negative regulation of cell fate specification, cellular components, including myosin complex and collagen, and molecular functions, such as extracellular matrix structural constituent, actin filament binding, and growth factor binding (Table 8, Figure 7A). At E17, downregulated DEGs were enriched in biological processes, such as cellular glucose homeostasis and negative regulation of tissue remodeling, cellular components, such as the ionotropic glutamate receptor complex and myelin sheath, and molecular functions such as GABA receptor binding and neuropeptide receptor binding (Table 9, Figure 7B).

At E20, upregulated DEGs were enriched in biological processes, such as cellular response to interferon-gamma, and regulation of cytokine production cellular components, such as keratin filament, and molecular functions, such as GTPase activity and cytokine receptor binding (Table 10, Figure 8A). At E20, downregulated DEGs were enriched in biological processes, such as regulation of the cellular ketone metabolic process and negative regulation of neurogenesis, cellular components, such as the extracellular matrix and neuronal cell body, and molecular functions, such as anion transmembrane transporter activity and voltage-gated ion channel activity (Table 11, Figure 8B).

The top 10 GO biological processes, cellular components, and molecular function enriched from DEGs that are upregulated or downregulated in MMC spinal cords, compared to the control at E15, E17, and E20, can be found in Figure S1. Several common GOs were identified when comparing MMC to either control group, but not when comparing control and vehicle groups (Figure S2). For example, at E15, both comparisons indicated that the biological process innervation and molecular function neurexin binding were downregulated. Additionally, at E17, both comparisons indicated a downregulation in neuronal action potential propagation and upregulation in the extracellular matrix. Finally, at E20, antigen processing and presentation of peptide or polysaccharide antigen via MHC class II and MHC class II protein complex were upregulated and myelination was downregulated.

### 3.3. KEGG Pathway Enrichment Analysis of Differentially Expressed Genes

**Figure 9:**
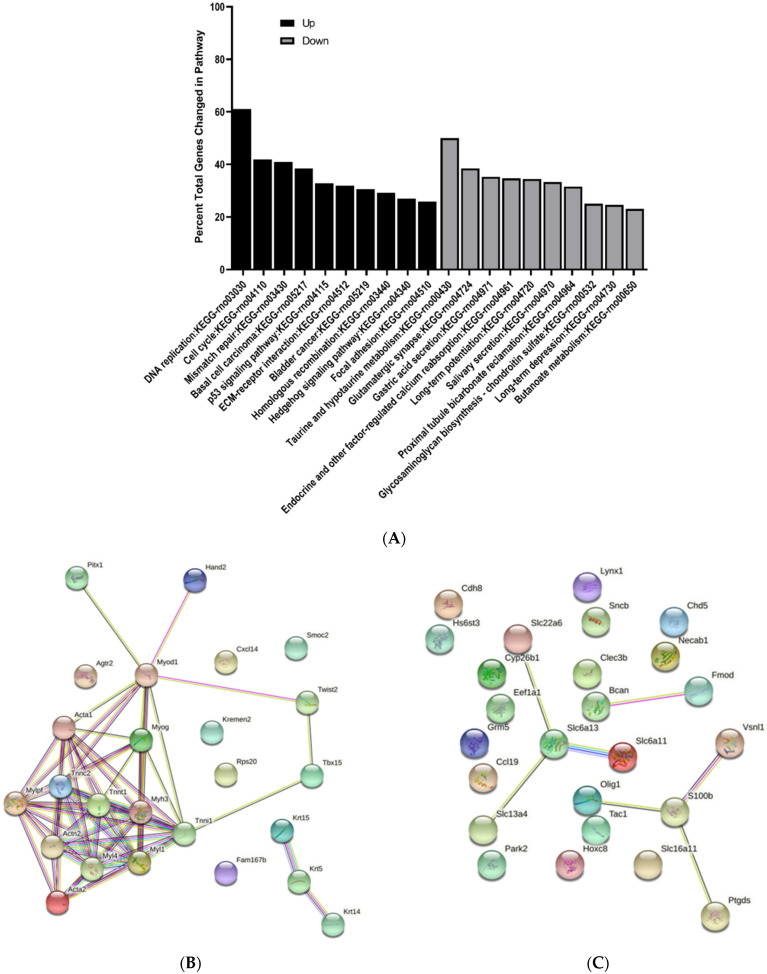
KEGG and protein–protein interaction network analysis of differentially expressed genes in the spinal cord of MMC and vehicle fetuses isolated at E15. (**A**) Top 10 KEGG pathways upregulated and downregulated as determined by percent total changed genes in each pathway. All pathways were greater than 1.5-fold change and *p* < 0.05. (**B**,**C**) Protein–protein interaction network analysis based on top 25 (**B**) upregulated and (**C**) downregulated differentially expressed genes.

**Figure 10:**
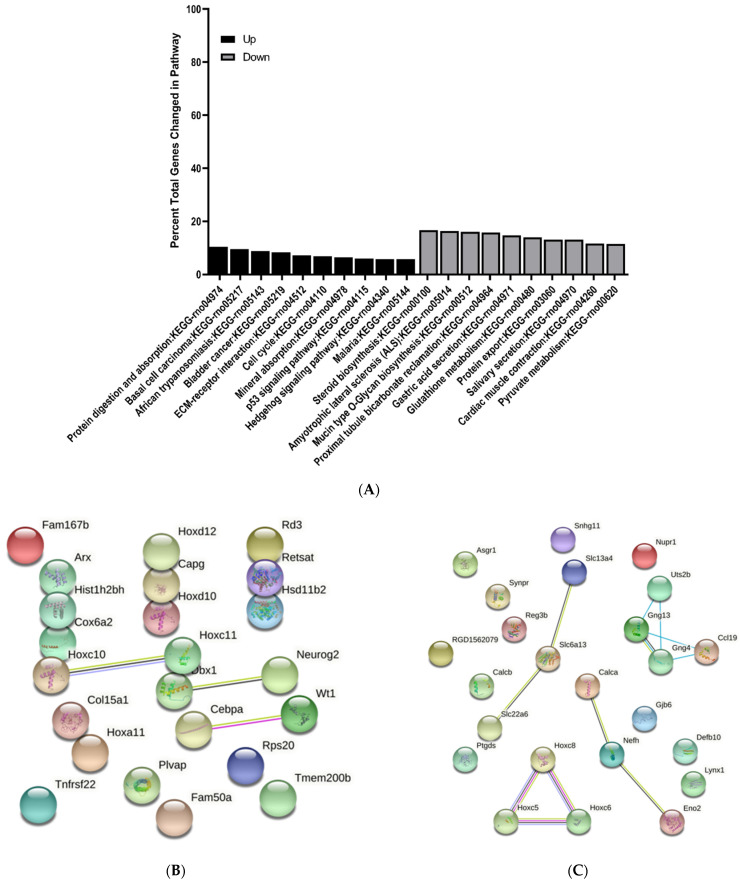
KEGG and protein–protein interaction network analysis of differentially expressed genes in the spinal cord of MMC and vehicle fetuses isolated at E17. (**A**) Top 10 KEGG pathways upregulated and downregulated as determined by percent total changed genes in each pathway. All pathways were greater than 1.5-fold change and *p* < 0.05. (**B**,**C**) Protein–protein interaction network analysis based on top 25 (**B**) upregulated and (**C**) downregulated differentially expressed genes.

**Figure 11:**
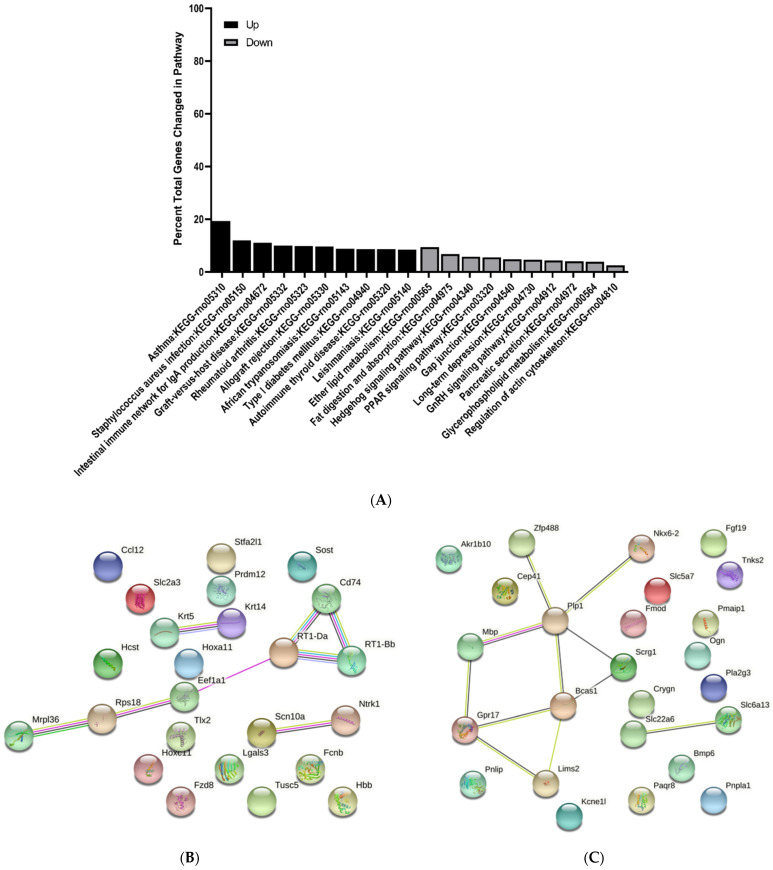
KEGG and protein–protein interaction network analysis of differentially expressed genes in the spinal cord of MMC and vehicle fetuses isolated at E20. (**A**) Top 10 KEGG pathways upregulated and downregulated as determined by percent total changed genes in each pathway. All pathways were greater than 1.5-fold change and *p* < 0.05. (**B**,**C**) Protein–protein interaction network analysis based on top 25 (**B**) upregulated and (**C**) downregulated differentially expressed genes.

The top 10 upregulated and downregulated KEGG pathways were determined and reported as percent total changed genes in each pathway. All pathways identified were greater than 1.5-fold change and *p* < 0.05. Interestingly, there were several common pathways changed at E15 and E17 when comparing MMC and vehicle groups (Figure 9A and Figure 10A). For example, at both time points, p53 signaling and hedgehog signaling were upregulated; however, fewer genes were changed in these pathways at E17 (Figure 10A). Additionally, at E17, metabolic pathways including glutathione and pyruvate metabolism were also downregulated and ECM receptor interaction was upregulated (Figure 10A). At E20, immunological pathways, such as staphylococcus aureus infection, were upregulated, while metabolic pathways including PPAR signaling, ether lipid metabolism and glycerophospholipid metabolism, were downregulated (Figure 11A). Additionally, at this time point, gap junction, and regulation of actin cytoskeleton were also downregulated (Figure 11A). 

The top 10 upregulated and downregulated KEGG pathways identified between the MMC and control groups and control and vehicle groups are found in Figures S3 and S4, respectively. Several common pathways were identified at each time point between MMC and either of the control groups. For example, the cell cycle and ECM receptor interactions were upregulated in both comparisons at E15 and E17. Furthermore, both comparisons demonstrated upregulation in autoimmune diseases, such as rheumatoid arthritis, type 1 diabetes mellitus, and autoimmune thyroid disease.

### 3.4. Protein–Protein Interaction Network Analysis of Differentially Expressed Genes

To investigate the interactions between DEGS, protein–protein interactions (PPI) were determined for each time point by submitting the top 25 up and downregulated DEGs between vehicle and MMC groups to the string. At E15, we identified a cluster of interactions between upregulated DEGs involved in structure and skeletal muscle development including Acta1, Actn2, and Myog (Figure 9B). Additionally, interactions between various downregulated transporters, such as Slc22a6 and Slc6a13, were determined as well as proteins involved in CNS cell fate determination, including Olig1 and S100b (Figure 9C); however, these interactions were also identified when comparing MMC groups to the control group (Figure S5). Furthermore, interactions between the same downregulated transporters were also identified at E17 in addition to proteins involved in neuronal cell identity, such as those in the Hox family, and neuron and axon maturation, including Calca and Nefh (Figure 10C). Interestingly, similar interactions were also identified when comparing MMC and control groups (Figure S6). Specific to the comparison between MMC and vehicle groups were the interactions between upregulated Dbx1 and neurog, both involved in neurogenesis, as well as Cebpa and Wt1, which are involved in the differentiation and survival of non-CNS cell types, including adipocytes and granulocytes (Figure 10B). Furthermore, interactions between downregulated Uts2b, Gng13, CCL19, and Gng4 were demonstrated (Figure 10C). At E20, PPI analysis indicated interactions between a host of downregulated DEGs involved myelination and oligodendrogenesis (Figure 11C). Similar to interactions observed at E17, the majority of these interactions were also found when comparing MMC and control groups (Figure S7); however, specific to the comparison of MMC and vehicle were the interactions between GPR17 and Bcas1 with Lims2 (Figure 11C). Interactions involving upregulated DEGs associated with MHC class II immune reactions, CD74 and RT1-Da were identified at E20 when comparing MMC and the control group (Figure S8B), with the addition of RT1-Bb and Eef1a1 in the comparison between MMC and vehicle groups (Figure 11B).

### 3.5. Validation by qRT-PCR

**Figure 12:**
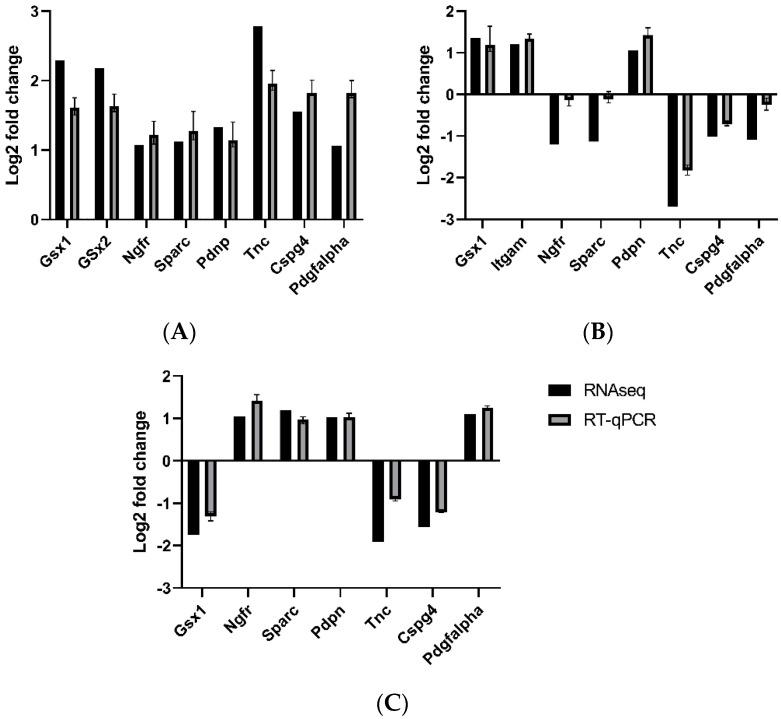
Comparison between RNA sequencing results and RT-qPCR between MMC and vehicle at (**A**) E15, (**B**) E17, and (**C**) E20.

To validate the sequencing results, the gene expression of 7–8 genes was randomly measured using qRT-PCR. The Log2fold change was determined between spinal cords isolated from MMC and vehicle groups at each time point and analyzed compared to results from the RNA sequencing (Figure 12). While the absolute values were not identical between each measurement type, the trend changes between each gestational time point were congruent with the results obtained from RNA sequencing. These data support that the RNA sequencing method provided reliable quantifications.

## 4. Discussion

In this study, we employed RNA sequencing to characterize the chronology of the most significant gene expression alterations in the spinal cord after defect development and exposure to the amniotic fluid in the congenital retinoic acid rat model of spina bifida. Models created from single or double mutants in mice do not always match the phenotype of human neural tube defect with the same mutation [12]. For example, a mutation in Vang-like protein 1 (VANGL1) was associated with neural tube defects in humans; however, there was not a neural tube defect phenotype found in homozygous mouse VANGL1 mutants [12]. It is suggested that human neural tube defects are a result of a combination of interacting mutations. The retinoic acid model is similar to human MMC both developmentally and anatomically, so this model may be also associated with interacting mutations [16]. Therefore, the use of retinoic acid is a translationally relevant model to study human MMC. By comparing the gene expression of spinal cords isolated from fetuses with retinoic acid-induced spina bifida to those of its control siblings or fetuses from vehicle-treated dames, we identified over 3000 differentially expressed genes (DEGs) at E15, over 1000 DEGs at E17, and over 300 DEGs at E20. Using this well-established and cost-efficient teratogenic animal model and the matched internal controls and vehicle controls allowed us to identify the changes during the second hit MMC pathophysiology (i.e., the acquired degenerative effect after amniotic fluid exposure) and not due to specific genetic mutations. We used this information to then predict the potential role of the DEGs using GO analysis, KEGG pathway analysis, and protein–protein interaction analysis at each time point. To our knowledge, this is the first study of its kind that identifies key DEGs and potential pathways that could be involved in the neural alterations of spina bifida in this model in utero. We propose that this information lays a foundation for the further study of novel pathways that could potentially be involved in the advancement of spina bifida and specific clinical outcomes. Additionally, these results may also lead to the future tailoring time-specific treatments in combination with the current standard of care that would potentially enhance current surgical spina bifida repair strategies as well as improve overall clinical outcomes. 

Our results suggest that a spina bifida defect is associated with alterations in the spinal cord gene expression that regulates aspects of cell survival and positioning, neuron function, and skeletal muscle development during E15 and E17 gestational points. Compared to fetuses with normal development, genes involved in p53 signaling were upregulated in fetuses with MMC at E15, a time point similar to gestational weeks 5–6 in humans [17]. This potential role of p53 signaling is supported by previous evidence that p53 mRNA is upregulated at E15 in the spinal cord of rat fetuses with retinoic acid-induced spina bifida [18]. This pathway is likely involved in the extreme apoptosis found in the spinal cord during spina bifida, which consequently leads to neuronal cell loss, contributing to the impairment of neurological functions, such as motor skills [8,18,19]. Similarly, KEGG pathway analysis also predicted hedgehog signaling to be upregulated, which was previously implicated in neural tube defects [4]. Abnormal hedgehog signaling decreases the survival of neuronal precursors and alters the position of motor neurons resulting in abnormalities in the structure of the motor column [20,21]. Motor neuron function may also potentially be impacted through hedgehog signaling effects on cellular retinoic acid-binding protein 1 (CRABP1) in which variants were observed in patients with neural tube defects [22,23]. Additionally, the glutamate receptor complex was downregulated at this time point, which also diminishes neural cell migration [24]. Furthermore, our results support modifications in neuron function potentially due to downregulation in synaptic vesicle maturation and GABA receptor binding, which plays a role in synapse formation [25]. Finally, DEGs involved in skeletal muscle development and cytoskeleton were upregulated at this time point. Previous studies support the accumulation of actomyosin machinery, which increases tissue stiffness and mechanically inhibits the normal closure of the neural plate, leading to a neural tube defect [26,27,28].

Interestingly, p53 and hedgehog signaling were also upregulated in fetuses with retinoic acid-induced MMC at E17, a time point similar to gestational weeks 20–26 in humans [17]. This was also associated with the downregulation of the glutamate receptor complex, supporting that neuronal survival and positioning continued to be impacted during gestation of this model. However, during this time point, further alterations were observed that diminish neuron function and may lead to clinical outcomes such as paralysis and neurological dysfunction. For example, downregulations in metabolic processes, such as those described by GOs, such as glutathione metabolism, pyruvate metabolism, and cellular glucose homeostasis, were also evident. Deficiencies in molecules involved in glutathione, a major antioxidant, metabolism have been linked to neuronal cell loss in the brain and cognitive impairment, a significant clinical outcome in spina bifida patients [29,30]. In addition, abnormalities in glucose metabolism may lead to impairments in neuron function, as neurons not only break down glucose to meet energetic demands, but they also consume pyruvate that is released by astrocytes after glycolysis [31,32]. Pre-clinical and clinical studies indicate that diabetic mothers or mothers with alterations in genes that regulate glucose metabolism have an increased risk of neural tube defects, supporting a potential role for glucose metabolism in the progression of spina bifida [33,34]. Furthermore, at E17, GO analysis also indicated downregulation of processes involved in myelin sheath production, which is heavily controlled by oligodendrocytes [35]. This effect is likely due to dysregulation in hedgehog signaling at E15 and E17, as this pathway is a major regulator of oligodendrocyte differentiation and function [36].

The dysregulation of processes involved in metabolic pathways and myelination was also apparent in fetuses with retinoic acid-induced MMC at E20, a time point similar to gestational week 34 in humans [17]. In contrast to glutathione and glucose metabolism downregulated at E17, KEGG analysis indicated the downregulation of pathways involved in lipid metabolism, such as lipase activity, ether lipid metabolism, and glycerophospholipid metabolism later in gestation. While few studies have investigated a role for lipid metabolism in spina bifida progression, rare variants in lipid metabolism have been observed in patients with spina bifida phenotypes [37]. Additionally, modifications in this pathway have been connected to diseases associated with motor neuron loss, such as amyotrophic lateral sclerosis in which patients develop muscle paralysis [38]. Furthermore, while several DEGs involved in oligodendrogenesis and myelination were downregulated as a result of retinoic acid, but not associated with spina bifida, other such DEGs including Gpr17, Lims2, and Bcas1 were downregulated in the spinal cord of fetuses with MMC defects. These specific genes were also significantly reduced in prenatal neurosphere cultures after BMP2 treatment, indicating the potential role of BMP2 in diminishing myelination during the progression of spina bifida [39]. Finally, specific only to E20, our results provide evidence that inflammatory processes are upregulated in the spinal cord of fetuses with retinoic acid-induced MMC. Our previous data further support this claim, as an increase in activated microglia, characterized as Iba1+ and MHC class II+ cells, IL-1B, IL-6, and IFN-g, was observed in the spinal cord of fetuses with MMC at E20, but not at earlier time points [8].

We hypothesize that the changes in gene expression we observed after the spina bifida defect is at least in part due to exposure of the spinal cord to the amniotic fluid. Studies on amniotic fluid supernatant collected from pregnant women at the time of open defect identified alterations in pathways similar to our results including those associated with neuronal development, axonal development, and synapse formation [40]. Interestingly, the inflammation had some of the most prominent alterations [40]. Furthermore, this inflammatory response may be due to the toxic components of amniotic fluid, as the amniotic fluid of rats with retinoic acid-induced MMC contained higher amylase levels and activity compared to healthy controls [41]. While the specific role of amylase in spinal cord inflammation and spina bifida outcomes has not been investigated, serum amylase is elevated during pancreatitis and is associated with the elevation of pro-inflammatory cytokines, such as Il-1B and IL-6, which supports the potential pro-inflammatory role of amylase in amniotic fluid [42].

## 5. Conclusions

In conclusion, through a comprehensive time-course transcriptomic analysis, this is the first study that characterized the progressive changes that occur in the neural tissue after exposure to the amniotic fluid in utero, using the congenital retinoic acid-induced spina bifida rodent model. Current standard surgical strategies only structurally repair the defect and inhibit further neurological damage due to amniotic fluid exposure. Likely due to limited understanding of the mechanisms that drive spina bifida degeneration in utero, there are no treatments available that regenerate healthy neural tissue and function after spina bifida diagnosis. Our results provide evidence that different mechanisms may play more important roles during specific periods throughout fetal progression. Therefore, it may be beneficial to tailor new therapeutic strategies to the gestational age at the time of treatment as well as to entertain an approach where a combination of pathways is targeted. Furthermore, future studies further elucidating the specific targets at different gestational ages during disease progression should be a focus in the spina bifida research community.

## Figures and Tables

**Table 1:** TaqMan probes used for RT-qPCR analysis.

mRNA	Name	* Assay Code Number
Gsx1	GS Homeobox1	Rn01412792_g1
Gsx2	GS Homeobox2	Rn03810223_m1
Ngfr	Nerve Growth Factor Receptor	Rn00561634_m1
Sparc	Secreted Protein, Acidic, Cysteine-Rich	Rn01470624_m1
Pdpn	Podoplanin	Rn00571195_m1
Hprt1	Hypoxanthine Phosphoribosyl Transferase 1	Rn01527840_m1
Tnc	Tenascin C	Rn01454948_m1
Pdgfa	Platelet-Derived Growth Factor Polypeptide	Rn00709363_m1

* Probe codes from Thermo Fisher Scientific.

**Table 2:** Number of differentially expressed genes identified as >1.5-fold change and *p* < 0.05 and % of total differentially expressed genes up- or downregulated.

	E15			E17			E20		
	Total	Upregulated	Downregulated	Total	Upregulated	Downregulated	Total	Upregulated	Downregulated
MMC vs. Vehicle	3022	1324 (44%)	1698 (56%)	1167	312 (27%)	855 (73%)	302	133 (44%)	169 (56%)
MMC vs. Control	3777	2119 (56%)	1658 (44%)	1847	264 (14%)	1583 (86%)	469	154 (33%)	315 (67%)
Control vs. Vehicle	505	147 (29%)	358 (71%)	327	117 (56%)	210 (44%)	355	269 (76%)	86 (24%)

**Table 3:** Top 25 upregulated and downregulated genes between MMC and vehicle groups at E15.

Gene Symbol	Full Gene Name	Fold Change	*p* Value
Actc1	Actin, alpha cardiac muscle 1	68.26	$6.81\times 10^{-6}$
Tnni1	Troponin I, slow skeletal muscle	20.94	$3.828\times 10^{-5}$
Rps20	40S ribosomal protein S20	20.21	$1.408\times 10^{-3}$
Myh3	Myosin-3	17.56	$3.27\times 10^{-6}$
Tnnc2	Troponin C, skeletal muscle	15.43	$2.59\times 10^{-5}$
Krt5	Keratin, type II cytoskeletal 5	14.95	$6.67\times 10^{-3}$
Myl1	Myosin light chain 1/3, skeletal muscle isoform	14.81	$3.88\times 10^{-5}$
Myog	Myogenin	13.50	$1.30\times 10^{-5}$
Cxcl14	C-X-C motif chemokine 14 precursor	12.76	$4.99\times 10^{-4}$
Twist2	Twist-related protein 2	12.62	$5.29\times 10^{-6}$
Tnnt1	Troponin T, slow skeletal muscle	12.24	$5.33\times 10^{-5}$
Smoc2	SPARC-related modular calcium-binding protein 2 precursor	11.22	$4.24\times 10^{-6}$
Fam167b	Family with sequence similarity 167, member B	10.45	$6.29\times 10^{-4}$
Agtr2	Type-2 angiotensin II receptor	10.28	$1.89\times 10^{-4}$
Krt14	Keratin, type I cytoskeletal 14	10.22	$4.65\times 10^{-3}$
Myod1	Myoblast determination protein 1	10.01	$9.92\times 10^{-6}$
Kremen2	Kremen protein 2 precursor	9.82	$9.19\times 10^{-6}$
Myl4	Myosin light chain 4	9.67	$1.36\times 10^{-4}$
Pitx1	Pituitary homeobox 1	9.57	$9.11\times 10^{-4}$
Hand2	Heart- and neural crest derivatives-expressed protein 2	9.45	$3.02\times 10^{-4}$
Krt15	Keratin, type I cytoskeletal 15	9.23	$1.43\times 10^{-3}$
Mylpf	Myosin regulatory light chain 2, skeletal muscle isoform	8.91	$1.06\times 10^{-4}$
Actn2	Actinin alpha 2	7.50	$1.11\times 10^{-4}$
Acta1	Actin, alpha skeletal muscle	6.50	$2.38\times 10^{-5}$
Tbx15	T-box transcription factor TBX15	6.44	$5.78\times 10^{-4}$
EF1A	Elongation factor 1-alpha	−51.18	$4.81\times 10^{-3}$
Lynx1	Ly-6/neurotoxin-like protein 1 precursor	−13.24	$2.35\times 10^{-6}$
Necab1	N-terminal EF-hand calcium-binding protein 1	−8.35	$3.64\times 10^{-5}$
Slc6a13	Sodium- and chloride-dependent GABA transporter 2	−7.65	$2.54\times 10^{-5}$
Bcan	Brevican core protein isoform 1 precursor	−7.18	$3.16\times 10^{-5}$
Slc13a4	Solute carrier family 13 member 4 precursor	−7.01	$6.39\times 10^{-5}$
Clec3b	C-type lectin domain family 3, member b	-6.78	$7.60\times 10^{-5}$
Hoxc8	Homeobox protein Hox-C8	−6.15	$8.64\times 10^{-4}$
Slc6a11	Sodium- and chloride-dependent GABA transporter 3	−5.85	$2.47\times 10^{-5}$
S100b	Protein S100-B	−5.63	$1.49\times 10^{-4}$
Grm5	Metabotropic glutamate receptor 5	−5.40	$2.03\times 10^{-5}$
Slc22a6	Solute carrier family 22 member 6	−4.86	$1.65\times 10^{-4}$
Ptgds	Prostaglandin-H2 D-isomerase	−4.63	$6.46\times 10^{-5}$
Vsnl1	Visinin-like protein 1	−4.61	$1.87\times 10^{-4}$
Slc16a11	Solute carrier family 16, member 11	−4.26	$2.41\times 10^{-4}$
Ccl19	C-C motif chemokine 19 precursor	−4.20	$2.18\times 10^{-4}$
Park2	E3 ubiquitin-protein ligase parkin	−4.19	$7.30\times 10^{-5}$
Cdh8	Cadherin-8 precursor	−3.90	$3.29\times 10^{-4}$
Sncb	Beta-synuclein	−3.80	$6.87\times 10^{-4}$
Fmod	Fibromodulin precursor	−3.73	$4.79\times 10^{-4}$
Hs6st3	Heparan-sulfate 6-O-sulfotransferase 3 precursor	−3.72	$1.41\times 10^{-3}$
Cyp26b1	Cytochrome P450 26B1	−3.69	$1.18\times 10^{-4}$
Tac1	Protachykinin-1 Substance P Neurokinin A Neuropeptide K Neuropeptide gamma C-terminal-flanking peptide	−3.69	$2.52\times 10^{-3}$
Olig1	Oligodendrocyte transcription factor 1	−3.64	$1.10\times 10^{-3}$
Chd5	Chromodomain-helicase-DNA-binding protein 5	−3.63	$2.48\times 10^{-4}$

**Table 4:** Top 25 upregulated and downregulated genes between MMC and vehicle groups at E17.

Gene Symbol	Full Gene Name	Fold Change	*p* Value
Fam167b	Family with sequence similarity 167, member B	8.18	$1.12\times 10^{-2}$
Hoxc11	Homeobox C11	7.72	$6.76\times 10^{-3}$
Fam50a	Protein Fam50a	6.96	$9.29\times 10^{-3}$
Dbx1	Homeobox protein DBX1	5.82	$2.49\times 10^{-3}$
Capg	Macrophage-capping protein	5.59	$8.40\times 10^{-5}$
Hoxa11	Protein Hoxa10	5.40	$1.36\times 10^{-2}$
Tmem200b	Transmembrane protein 200B	4.16	$4.99\times 10^{-4}$
Cox6a2	Cytochrome c oxidase subunit 6A2, mitochondrial	4.06	$9.10\times 10^{-2}$
Hbb-b2	Hemoglobin subunit beta-2	3.97	$2.12\times 10^{-3}$
Plvap	Plasmalemma vesicle-associated protein	3.94	$2.05\times 10^{-2}$
Hoxd12	Homeo box D12	3.92	$1.36\times 10^{-4}$
Rd3	Retinal degeneration 3	3.67	$1.46\times 10^{-2}$
Neurog2	Protein Neurog2	3.67	$1.26\times 10^{-2}$
Retsat	All-trans-retinol 13,14-reductase precursor	3.41	$3.32\times 10^{-4}$
Tnfrsf22	Tumor necrosis factor superfamily member 22	3.37	$6.39\times 10^{-4}$
Rps20	40S ribosomal protein S20	3.18	$7.69\times 10^{-3}$
Hist1h2bc	Histone H2B	3.14	$1.93\times 10^{-3}$
Hoxc10	Homeo box C10	3.13	$2.03\times 10^{-3}$
Arx	Homeobox protein ARX	2.97	$5.39\times 10^{-3}$
ATP5F1E	ATP synthase subunit epsilon, mitochondrial	2.96	$2.81\times 10^{-3}$
Col15a1	Collagen alpha-1(XV) chain precursor	2.96	$4.95\times 10^{-2}$
Hsd11b2	Corticosteroid 11-beta-dehydrogenase isozyme 2	2.95	$3.39\times 10^{-4}$
Wt1	Wilms tumor protein homolog	2.90	$9.21\times 10^{-4}$
Hoxd10	Homeo box D10	2.82	$2.85\times 10^{-3}$
Cebpa	CCAAT/enhancer-binding protein alpha	2.81	$1.91\times 10^{-2}$
Ptgds	Prostaglandin-H2 D-isomerase	−49.27	$1.02\times 10^{-4}$
Gm10068	Chromobox 3, pseudogene 7	−11.56	5.10 × 10^−5^
Eno2	Enolase 2	−9.46	$2.43\times 10^{-3}$
Calca	Calcitonin	−6.91	$1.68\times 10^{-4}$
SDHAF1	Succinate dehydrogenase assembly factor 1	−6.07	$3.42\times 10^{-2}$
Defb10	Beta-defensin 10	-5.15	$1.42\times 10^{-4}$
Lynx1	Ly-6/neurotoxin-like protein 1 precursor	−4.83	$7.85\times 10^{-3}$
Reg3b	Regenerating islet-derived protein 3-beta	−4.55	$2.01\times 10^{-2}$
Slc22a6	Solute carrier family 22 member 6	−4.41	$9.99\times 10^{-5}$
Hoxc8	Homeobox protein Hox-C8	−4.35	$7.76\times 10^{-4}$
Hoxc5	Homeo box C5	−4.16	$1.38\times 10^{-2}$
Uts2d	Urotensin-2B	−4.06	$5.12\times 10^{-4}$
Slc13a4	Solute carrier family 13 member 4 precursor	−4.06	$1.15\times 10^{-3}$
Gng4	Guanine nucleotide-binding protein subunit gamma 4	−4.01	$7.08\times 10^{-3}$
NUPR1	Nuclear protein 1	−3.83	$3.09\times 10^{-3}$
Slc6a13	Sodium- and chloride-dependent GABA transporter 2	−3.82	$2.29\times 10^{-4}$
Nefh	Neurofilament heavy polypeptide	−3.73	$8.48\times 10^{-4}$
Calcb	Calcitonin gene-related peptide 2 precursor	−3.62	$1.87\times 10^{-4}$
Asgr1	Asialoglycoprotein receptor 1	−3.60	$2.10\times 10^{-4}$
Gng13	Guanine nucleotide binding protein, gamma 13	−3.48	$3.79\times 10^{-3}$
Ccl19	C-C motif chemokine 19 precursor	−3.44	$5.99\times 10^{-3}$
Hoxc6	Homeo box C6	−3.33	$2.14\times 10^{-3}$
Snhg11	Small nucleolar RNA host gene 11 (non-protein coding)	−3.32	$6.00\times 10^{-3}$
Gjb6	Gap junction beta-6 protein	−3.30	$4.40\times 10^{-3}$
Synpr	Synaptoporin	−3.29	$1.24\times 10^{-3}$

**Table 5:** Top 25 upregulated and downregulated genes between MMC and vehicle groups at E20.

Gene Symbol	Full Gene Name	Fold Change	*p* Value
EF1A	Elongation factor 1-alpha	129.91	$6.48\times 10^{-5}$
Krt5	Keratin, type II cytoskeletal 5	51.61	$2.60\times 10^{-2}$
Hoxc11	Homeobox C11	29.97	$1.27\times 10^{-2}$
HBB	Hemoglobin subunit beta-2	29.46	$1.84\times 10^{-2}$
Krt14	Keratin, type I cytoskeletal 14	25.30	$2.63\times 10^{-2}$
Hoxa11	Homeobox A11	9.35	$3.12\times 10^{-3}$
RPS18	40S ribosomal protein S18	8.87	$1.14\times 10^{-3}$
Hoxa11	Protein Hoxa10; RCG52455	8.13	$3.06\times 10^{-3}$
RT1-Da	RT1 class II, locus Da precursor	6.32	$2.27\times 10^{-3}$
MRPL36	Mitochondrial ribosomal protein L36	5.85	$4.45\times 10^{-2}$
Ntrk1	High affinity nerve growth factor receptor	5.70	$4.95\times 10^{-3}$
Lgals3	Galectin-3	5.36	$2.53\times 10^{-2}$
Cd74	H-2 class II histocompatibility antigen gamma chain	5.23	$2.36\times 10^{-3}$
FZD8	Frizzled 8	4.84	$7.65\times 10^{-3}$
Prdm12	PR domain zinc finger protein 12	4.82	$8.55\times 10^{-3}$
Tlx2	T-cell leukemia, homeobox 2	4.82	$2.27\times 10^{-2}$
Stfa2l1	Stefin-3	4.70	$2.25\times 10^{-2}$
Ccl12	Chemokine (C-C motif) ligand 12 precursor	4.52	$1.95\times 10^{-2}$
Hcst	Hematopoietic cell signal transducer	4.50	$1.11\times 10^{-2}$
Tusc5	Tumor suppressor candidate 5 homolog	4.48	$1.28\times 10^{-2}$
Scn10a	Sodium channel protein type 10 subunit alpha	4.34	$6.80\times 10^{-3}$
Slc2a3	Solute carrier family 2, facilitated glucose transporter member 3	4.33	$2.77\times 10^{-3}$
RT1-Bb	Rano class II histocompatibility antigen, B-1 beta chain precursor	4.18	$1.38\times 10^{-3}$
Fcnb	Ficolin-2	4.04	$4.42\times 10^{-2}$
Sost	Sclerostin	4.03	$3.39\times 10^{-2}$
Pla2g3	Group 3 secretory phospholipase A2 precursor	−9.01	$1.47\times 10^{-2}$
Bcas1	Breast carcinoma-amplified sequence 1 homolog	−5.78	$3.52\times 10^{-2}$
Mbp	Myelin basic protein S	−5.72	$1.68\times 10^{-3}$
Tnks2	Tankyrase 2	−5.59	$5.93\times 10^{-3}$
Gpr17	Uracil nucleotide/cysteinyl leukotriene receptor	−5.58	$4.84\times 10^{-2}$
TSGA14	Testis-Specific Gene A14 Protein	−5.56	$6.59\times 10^{-3}$
Slc22a6	Solute carrier family 22 member 6	−5.19	$1.73\times 10^{-2}$
Pnlip	Pancreatic triacylglycerol lipase precursor	−4.83	$2.51\times 10^{-3}$
Slc6a13	Sodium- and chloride-dependent GABA transporter 2	−4.48	$3.47\times 10^{-3}$
Ogn	Osteoglycin	−4.46	$4.11\times 10^{-2}$
Paqr8	Membrane progestin receptor beta	−4.44	$2.86\times 10^{-2}$
Nkx6-2	NK6 homeobox 2	−4.35	$1.94\times 10^{-2}$
Crygn	Gamma-crystallin N	−4.23	$4.27\times 10^{-2}$
Scrg1	Scrapie-responsive protein 1	−4.09	$1.45\times 10^{-2}$
Plp1	Myelin proteolipid protein	−3.92	$5.92\times 10^{-3}$
Zfp488	Zinc Finger Protein 488	−3.89	$4.68\times 10^{-2}$
Fmod	Fibromodulin precursor	−3.80	$4.67\times 10^{-3}$
Pmaip1	Phorbol-12-myristate-13-acetate-induced protein 1	−3.79	$2.61\times 10^{-2}$
AKR1B10	Aldo-keto reductase family 1, member B10	−3.56	$4.93\times 10^{-2}$
Fgf15	Fibroblast growth factor 15 precursor	−3.50	$1.21\times 10^{-2}$
Slc5a7	High affinity choline transporter 1	−3.47	$3.53\times 10^{-2}$
Bmp6	Bone morphogenetic protein 6	−3.46	$2.36\times 10^{-2}$
Lims2	LIM and senescent cell antigen-like-containing domain protein 2	−3.30	$3.69\times 10^{-2}$
Pnpla1	Patatin-like phospholipase domain-containing protein 1	−3.29	$4.12\times 10^{-2}$
Kcne1l	Potassium voltage-gated channel subfamily E member 1-like protein	−3.24	$6.35\times 10^{-3}$

**Table 6:** Top 10 GO biological processes, cellular components, and molecular functions enriched from differentially expressed genes that are upregulated in MMC spinal cords compared to vehicle at E15. All GO pathways were >1.5-fold and *p* < 0.05.

GO Biological Process	GO Cellular Component	GO Molecular Function
Regulation of retroviral genome replication	Cleavage furrow	Extracellular matrix binding
DNA ligation	Ribonucleoside-diphosphate reductase complex	Single-stranded DNA-dependent ATPase activity
Tight junction assembly	DNA polymerase complex	Peptidase activator activity
Wound healing, spreading of cells	T-tubule	Histone kinase activity
DNA biosynthetic process	Contractile fiber	Caspase regulator activity
Cytokinesis	Contractile fiber part	Ribonucleoside-diphosphate reductase activity
Regulation of mesoderm development	Perinuclear region of cytoplasm	Flap endonuclease activity
Glial cell migration	Extracellular vesicular exosome	Cyclin-dependent protein kinase activity
Memory	Cyclin-dependent protein kinase holoenzyme complex	DNA N-glycosylase activity
Negative regulation of cytokine production	Nuclear envelope	Receptor signaling protein tyrosine kinase activity

**Table 7:** Top 10 GO biological processes, cellular components, and molecular functions enriched from differentially expressed genes that are downregulated in MMC spinal cords compared to vehicle at E15. All GO pathways were >1.5-fold and *p* < 0.05.

GO Biological Process	GO Cellular Component	GO Molecular Function
Regulation of gamma-aminobutyric acid secretion	Sodium	Neurotransmitter binding
Positive regulation of epidermal growth factor receptor activity	Neuronal cell body membrane	Protein kinase C activity
Negative regulation of leukocyte migration	cAMP-dependent protein kinase complex	Adenylate cyclase activity
Protein localization to synapse	Heterotrimeric G-protein complex	Calcium-dependent protein kinase regulator activity
Regulation of ARF protein signal transduction	Periplasmic space	GABA receptor binding
Synaptic vesicle maturation	Protein phosphatase type 2A complex	Long-chain fatty acid-CoA ligase activity
Positive regulation of amine transport	Ionotropic glutamate receptor complex	Neurexin binding
Negative regulation of insulin receptor signaling pathway	Synapse part	Serotonin receptor activity
Cell junction maintenance	Recycling endosome membrane	Calcium-dependent protein binding
Innervation	Cell junction	Protein phosphatase regulator activity

**Table 8:** Top 10 GO biological processes, cellular components, and molecular functions enriched from differentially expressed genes that are upregulated in MMC spinal cords compared to vehicle at E17. All GO pathways were >1.5-fold and *p* < 0.05.

GO Biological Process	GO Cellular Component	GO Molecular Function
Female pregnancy	Cortical cytoskeleton	mRNA 3′-UTR binding
Proximal/distal pattern formation	Myosin complex	Extracellular matrix structural constituent
Specification of loop of Henle identity	Stress fiber	Growth factor binding
Regulation of endocrine process	Nucleosome	Manganese ion binding
Platelet activation	Collagen	Protein dimerization activity
Somatic stem cell maintenance	Basement membrane	Hormone activity
Multicellular organism adhesion	Extracellular matrix	C2H2 zinc finger domain binding
Retinol metabolic process	Hemoglobin complex	Oxygen transporter activity
Endochondral ossification	Extracellular space	Oxygen binding
Negative regulation of cell fate specification	Nucleus	Actin filament binding

**Table 9:** Top 10 GO biological processes, cellular components, and molecular functions enriched from differentially expressed genes that are downregulated in MMC spinal cords compared to vehicle at E17. All GO pathways were >1.5-fold and *p* < 0.05.

GO Biological Process	GO Cellular Component	GO Molecular Function
Regulation of response to food	Heterotrimeric G protein complex	Neuropeptide hormone activity
Gastric acid secretion	Perikaryon	Channel inhibitor activity
Negative regulation of tissue remodeling	Extrinsic to organelle membrane	GABA receptor binding
Neuronal action potential propagation	Endomembrane system	Retinoid binding
Peptide transport	Dendritic shaft	Neuropeptide receptor binding
Diol metabolic process	Axon part	Cyclic nucleotide binding
Regulation of cellular amine metabolic process	Myelin sheath	Tau protein binding
Regulation of glomerular filtration	Cell body	L amino acid transmembrane transporter activity
Negative regulation of muscle contraction	Extracellular region	Caspase regulator activity
Cellular glucose homeostasis	Ionotropic glutamate receptor complex	Oxidoreductase activity

**Table 10:** Top 10 GO biological processes, cellular components, and molecular functions enriched from differentially expressed genes that are upregulated in MMC spinal cords compared to vehicle at E20. All GO pathways were >1.5-fold and *p* < 0.05.

GO Biological Process	GO Cellular Component	GO Molecular Function
Mechanical stimulus involved in sensory perception of pain	Lytic vacuole	Sodium channel activity
Antigen processing and presentation of antigen via MHC class II	External side of plasma membrane	Cytokine receptor binding
Cellular response to interferon-gamma	MHC class II protein complex	GTPase activity
Sodium ion transport	Integral to plasma membrane	Cytokine activity
Positive regulation of antigen processing and presentation	Vacuolar membrane	Sequence specific DNA binding
Regulation of cytokine production involved in immune response	Nucleosome	Sequence specific DNA binding transcription factor activity
Negative regulation of leukocyte activation	Cell surface	Ligand-gated ion channel activity
Regulation of T cell differentiation	Intracellular	Heme binding
Negative regulation of protein complex assembly	Extracellular region part	Structure specific DNA binding
Regulation of alpha-beta T cell activation	Keratin filament	Oxygen transporter activity

**Table 11:** Top 10 GO biological processes, cellular components, and molecular functions enriched from differentially expressed genes that are downregulated in MMC spinal cords compared to vehicle at E17. All GO pathways were >1.5-fold and *p* < 0.05.

GO Biological Process	GO Cellular Component	GO Molecular Function
Response to toxin	Integral to plasma membrane	Carboxylic ester hydrolase activity
Myelination	Extracellular region	Carboxylic acid transmembrane transporter activity
Sterol transport	Caveola	Cytokine receptor binding
Regulation of symbiosis	Extracellular space	Amine transmembrane transporter activity
Negative regulation of neurogenesis	Extracellular matrix part	Voltage-gated ion channel activity
Regulation of cellular ketone metabolic process	Neuronal cell body	Secondary active transmembrane transporter activity
Regulation of steroid metabolic process	Extracellular matrix	Inorganic cation transmembrane transporter activity
Membrane organization	Nuclear membrane	Anion transmembrane transporter activity
Positive regulation of signal transduction		Cytokine activity
Regulation of lipid transport		

## Data Availability

RNAseq data were deposited in the Sequence Read Archive (SRA) and can be found via BioProject (PRJNA683230) and SRA(PRJNA683793).

## Supplementary Materials

The following are available online at https://www.mdpi.com/article/10.3390/brainsci11121593/s1, Figure S1: Top 10 GO biological processes, cellular components, and molecular functions enriched from DEGs that are upregulated (A–C) or downregulated (D–F) in MMC spinal cords compared to control at E15 (A,D), E17 (B,E), and E20 (C,F). Figure S2: Top 10 GO biological processes, cellular components, and molecular functions enriched from DEGS that are upregulated (A–C) or downregulated (D–F) in vehicle spinal cords compared to control at E15 (A,D), E17 (B,E), and E20 (C,F). Figure S3: Top 10 KEGG pathways upregulated and downregulated between MMC and control at E15 (A), E17 (B), and E20 (C) as determined by percent total changed genes in each pathway. Figure S4: Top 10 KEGG pathways upregulated and downregulated between vehicle and control at E15 (A), E17 (B), and E20 (C) as determined by percent total changed genes in each pathway. Figure S5: Protein–protein interaction network analysis based on top 25 downregulated (A) and upregulated (B) differentially expressed genes between MMC and control at E15. Figure S6: Protein–protein interaction network analysis based on top 25 downregulated (A) and upregulated (B) differentially expressed genes between MMC and control at E17. Figure S7: Protein–protein interaction network analysis based on top 25 downregulated (A) and upregulated (B) differentially expressed genes between MMC and control at E20. Figure S8: Protein–protein interaction network analysis based on top 25 downregulated (A) and upregulated (B) differentially expressed genes between vehicle and control at E15. Figure S9: Protein–protein interaction network analysis based on top 25 downregulated (A) and upregulated (B) differentially expressed genes between vehicle and control at E17. Figure S10: Protein–protein interaction network analysis based on top 25 downregulated (A) and upregulated (B) differentially expressed genes between vehicle and control at E20. Table S1: Top 25 upregulated and downregulated genes between MMC and control groups at E15. Table S2: Top 25 upregulated and downregulated genes between MMC and control groups at E17. Table S3: Top 25 upregulated and downregulated genes between MMC and control groups at E20. Table S4: Top 25 upregulated and downregulated genes between control and vehicle groups at E15. Table S5: Top 25 upregulated and downregulated genes between control and vehicle groups at E17. Table S6: Top 25 upregulated and downregulated genes between control and vehicle groups at E20. Graphical Abstract (created with BioRender.com).
