# Supplementary material for: Time Course Transcriptome Analysis of Spina Bifida Progression in Fetal Rats
Source: Brain Sci. 2021 Nov 30;11(12):1593. doi: 10.3390/brainsci11121593 (PMC8699677; doi:10.3390/brainsci11121593)
Supplement: Supplementary file 1 [file brainsci-11-01593-s001.zip › brainsci-1456669-supplementary.pdf]

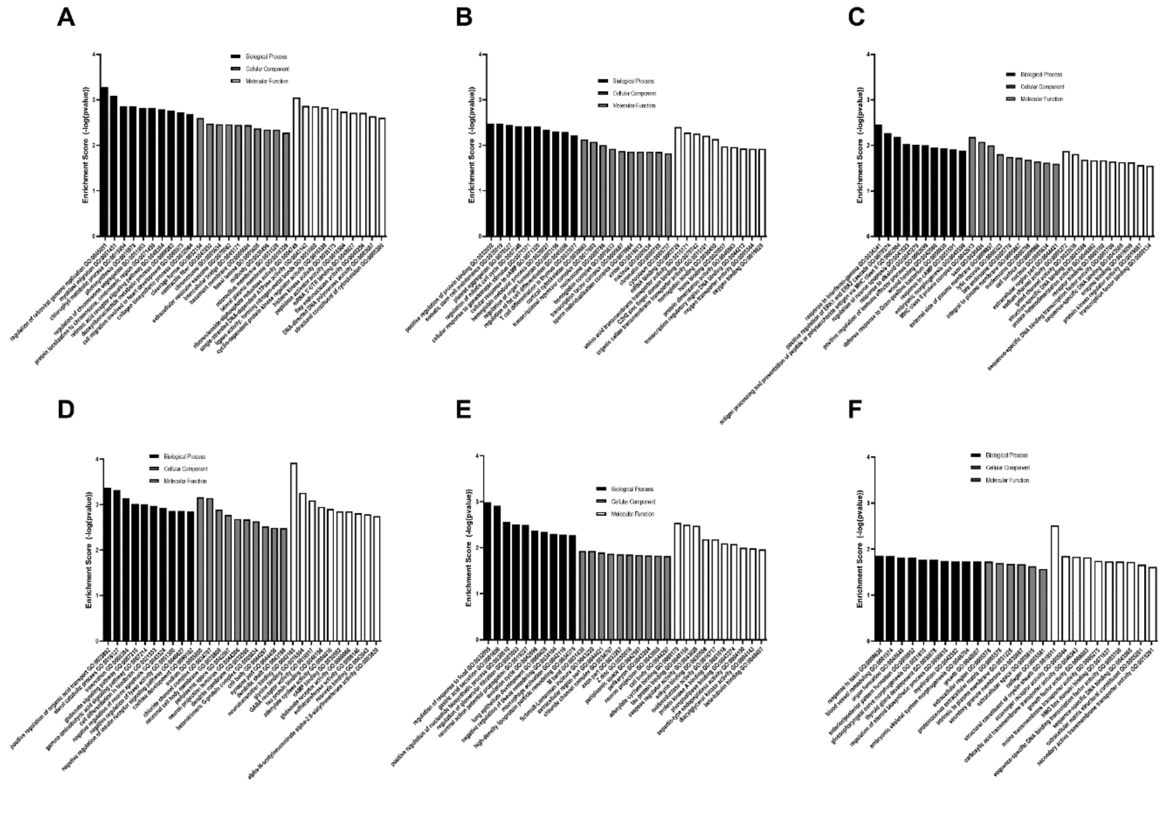

Figure S1: Top 10 GO biological processes, cellular components, and molecular functions enriched from DEGS that are upregulated (A-C) or downregulated (D-F) in MMC spinal cords compared to control at E15 (A,D), E17 (B,E), and E20 (C,F).

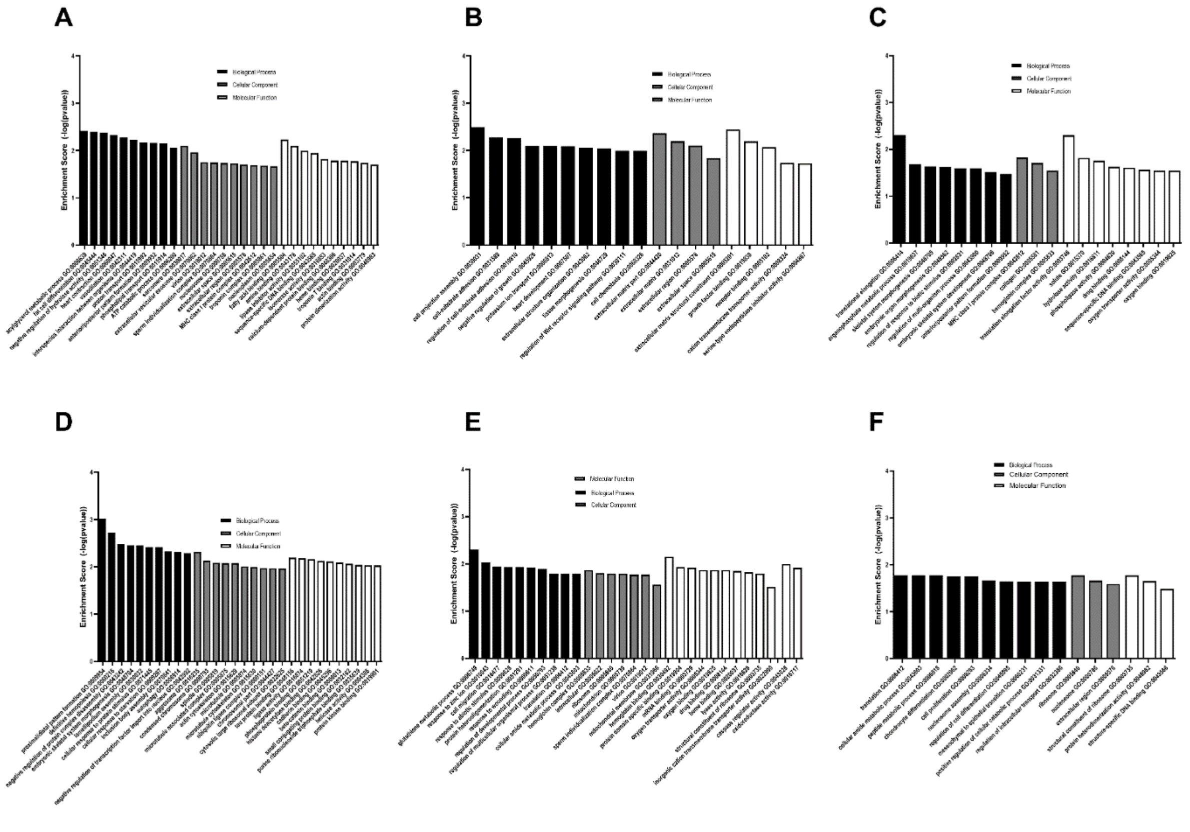

Figure S2: Top 10 GO biological processes, cellular components, and molecular functions enriched from DEGS that are upregulated (A-C) or downregulated (D-F) in vehicle spinal cords compared to control at E15 (A,D), E17 (B,E), and E20 (C,F).

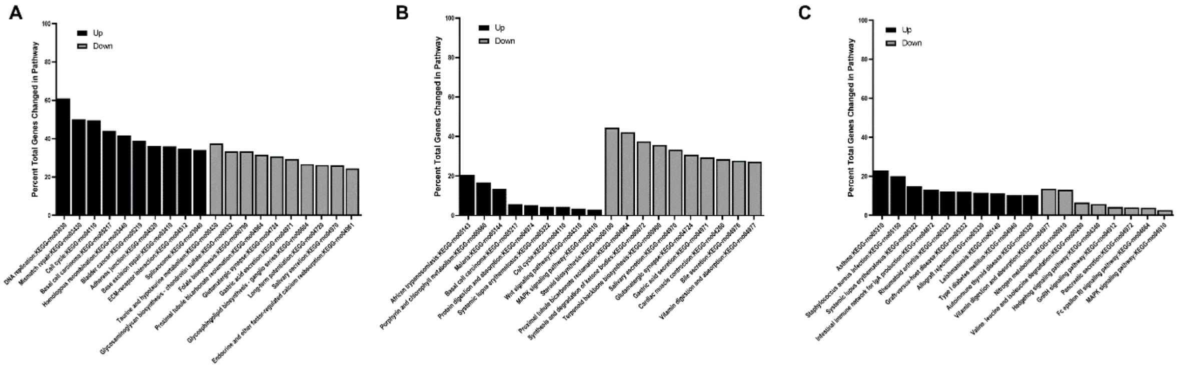

Figure S3: Top 10 KEGG pathways upregulated and downregulated between MMC and control at E15 (A), E17 (B), and E20 (C) as determined by percent total changed genes in each pathway.

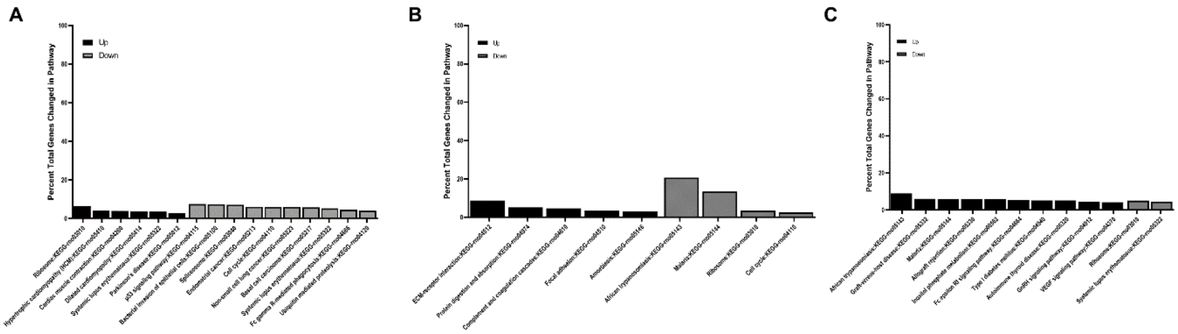

Figure S4: Top 10 KEGG pathways upregulated and downregulated between vehicle and control at E15 (A), E17 (B), and E20 (C) as determined by percent total changed genes in each pathway.

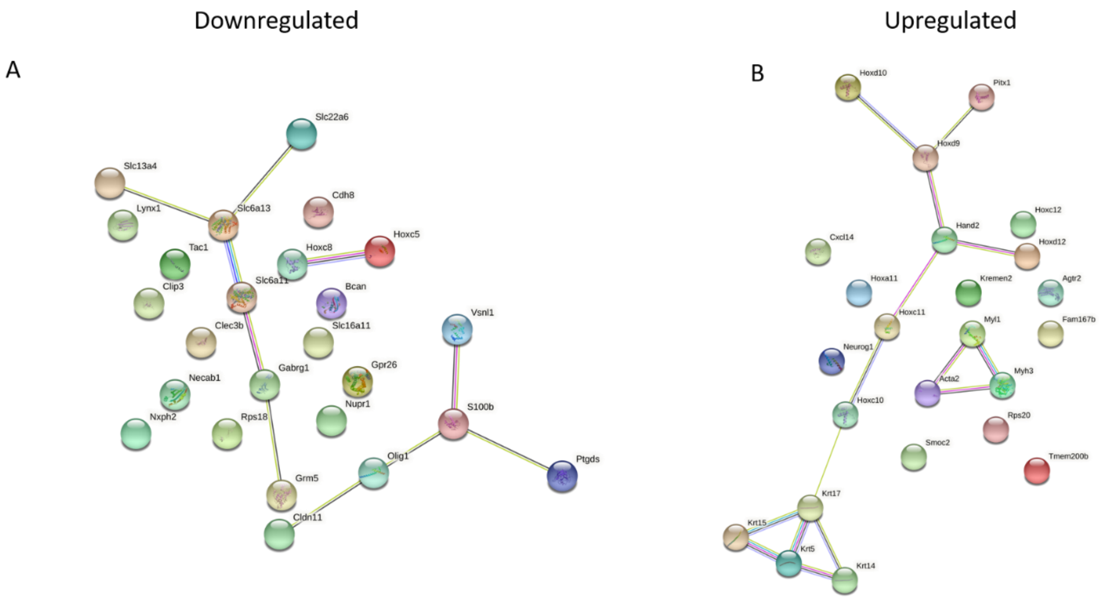

Figure S5: Protein-protein interaction network analysis based on top 25 downregulated (A) and upregulated (B) differentially expressed genes between MMC and control at E15.

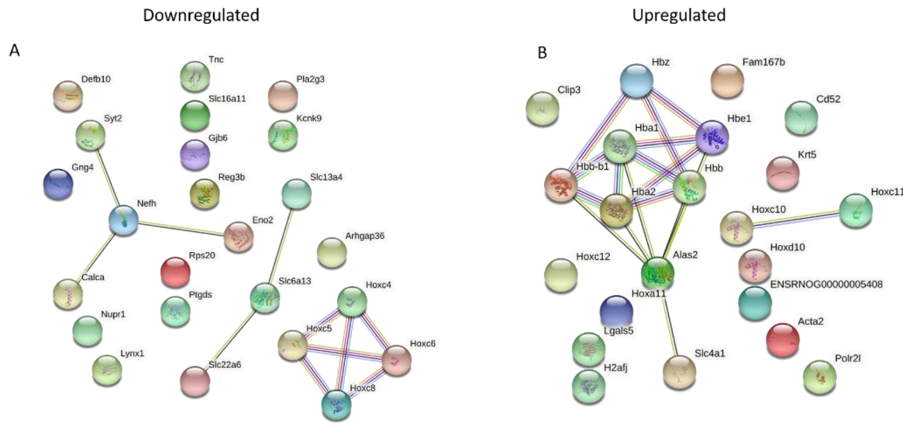

Figure S6: Protein-protein interaction network analysis based on top 25 downregulated (A) and upregulated (B) differentially expressed genes between MMC and control at E17.

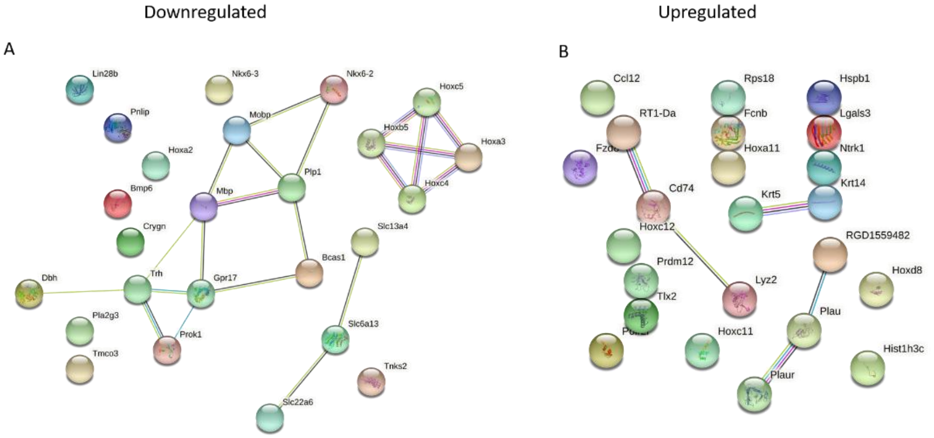

Figure S7: Protein-protein interaction network analysis based on top 25 downregulated (A) and upregulated (B) differentially expressed genes between MMC and control at E20.

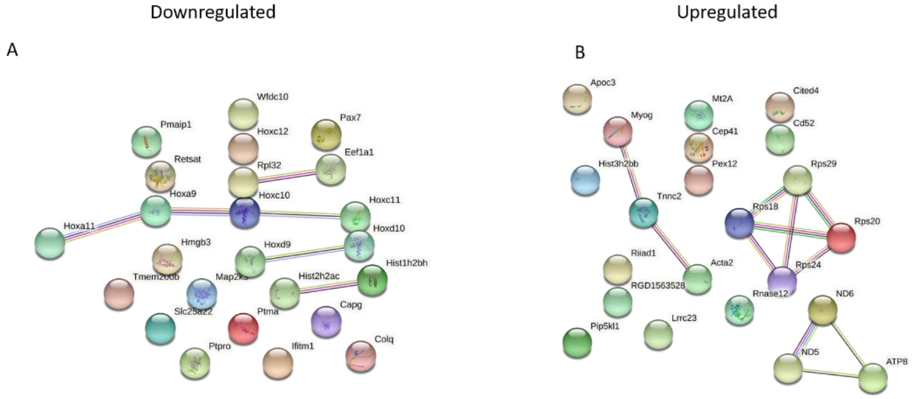

Figure S8: Protein-protein interaction network analysis based on top 25 downregulated (A) and upregulated (B) differentially expressed genes between vehicle and control at E15.

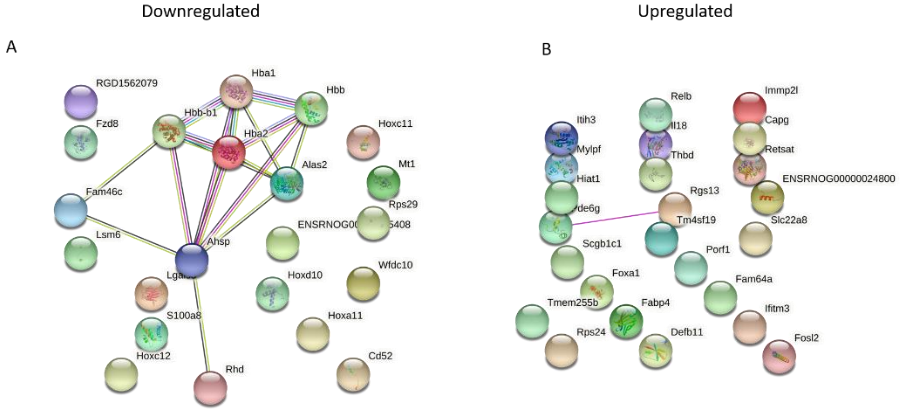

Figure S9: Protein-protein interaction network analysis based on top 25 downregulated (A) and upregulated (B) differentially expressed genes between vehicle and control at E17.

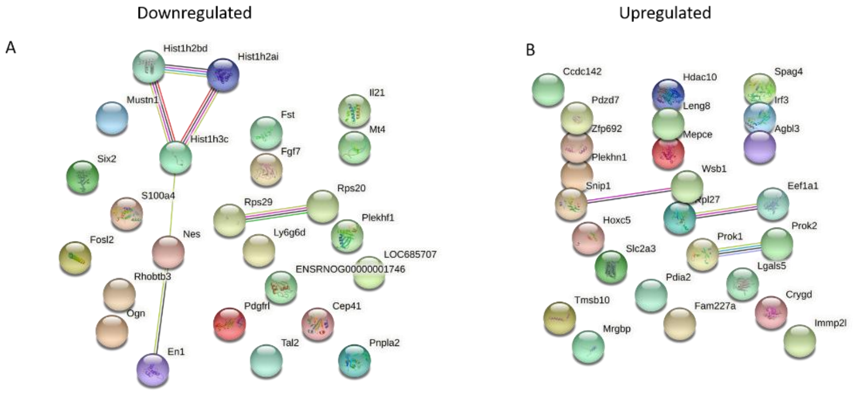

Figure S10: Protein-protein interaction network analysis based on top 25 downregulated (A) and upregulated (B) differentially expressed genes between vehicle and control at E20.

Table S1. Top 25 upregulated and downregulated genes between MMC and control groups at E15.

Gene Symbol	Full Gene Name	Fold Change	P value
Hoxc10	Homeobox C10	103.61	6.35E-04
Hoxc11	Homeobox C11	79.38	1.27E-04
Hoxc12	Homeobox C12	74.09	8.39E-03
Hoxd10	Homeobox D10	67.05	8.20E-06
Hoxd9	Homeobox D9	36.56	9.35E-04
Krt5	Keratin, type II cytoskeletal 5	14.25	9.20E-03
Hoxa11	Homeobox A11	13.10	7.74E-04
Actc1	Actin, alpha cardiac muscle 1	12.77	9.60E-04
Fam167b	Family with sequence similarity 167, member B	11.62	1.57E-04
Krt14	Keratin, type 1 cytoskeletal 14	11.52	2.54E-03
Neurog1	Neurogenin-1	11.17	3.79E-04
Myh3	Myosin-3	10.61	1.45E-04
Agtr2	Type-2 angiotensin II receptor	10.48	5.51E-04
Cxcl14	C-X-C motif chemokine 14 precursor	9.75	8.08E-04
Hand2	Heart-and neural crest derivatives expressed protein 2	9.45	3.04E-04
Rps20	40S ribosomal protein S20	9.14	4.66E-03
Pitx1	Pituitary homeobox 1	8.63	1.12E-03
Myl1	Mysoin light chain 1/3, skeletal muscle isoform	8.30	1.42E-03
Smoc2	SPARC-related modular calcium-binding protein 2 precursor	8.29	4.39E-05
Kremen2	kremen protein 2 precursor	8.16	5.95E-04
Hoxd12	homeo box D12	7.58	1.65E-02
Krt15	Keratin, type I cytoskeletal 15	7.58	2.82E-03
Hoxa11	Protein Hoxa10	7.36	1.91E-02
Tmem200b	transmembrane protein 200b	7.30	1.71E-04
Krt17	Keratin, type I cytoskeletal 17	6.97	5.95E-04
Clip3	Protein Clip3	-11.02	1.15E-02
Bcan	Brevican core protein isoform 1 precursor	-10.51	1.98E-06
Clec3b	C-type lectin domain family 3, member b	-9.94	2.03E-06
S100b	Protein S100-B	-9.76	1.01E-05
Lynx1	Ly-6/neurotoxin like protein 1 precursor	-8.44	4.39E-06
Slc6a13	Sodium and chloride dependent GABA transporter 2	-8.22	4.51E-06

Necab1	N-terminal EF-hand calcium binding protein 1	-7.76	5.64E-05
Ptgds	Prostaglandin-H2 D isomerase	-6.99	2.06E-05
Slc13a4	Solute carrier family 13 member 4 precursor	-6.91	8.56E-04
Slc6a11	Sodium and chloride dependent GABA transporter 3	-6.05	7.65E-06
Hoxc8	Homeobox protein Hox-C8	-5.80	9.63E-04
Grm5	Metabotropic glutamate receptor 5	-5.75	6.46E-06
Vsnl1	Visinin-like protein 1	-5.32	7.08E-05
RPS18	40s ribosomal protein S18	-5.31	2.89E-02
Cldn11	Claudin-11	-5.18	2.84E-05
Slc16a11	Solute carrier family 16, member 11	-5.02	1.13E-05
Slc22a6	Solute carrier family 22 member 6	-4.83	1.48E-03
Hoxc5	Homeo box C5	-4.81	8.88E-05
Nxph2	Neurexophilin	-4.63	4.11E-04
Tac1	Protachykinin precursor 1	-4.54	1.32E-03
Gabrg1	Gamma-aminobutyric acid receptor	-4.51	5.14E-04
Olig1	Oligodendrocyte transcription factor 1	-4.50	1.79E-04
NUPR1	Nuclear protein 1	-4.41	4.56E-04
CDh8	Cadherin-8 precursor	-4.41	2.36E-04
Gpr26	G-protein coupled receptor 26	-4.35	1.77E-05

Table S2. Top 25 upregulated and downregulated genes between MMC and control groups at E17.

Gene Symbol	Full Gene Name	Fold Change	P value
Hoxc12	homeobox protein Hox-C12	148.87	5.58E-03
Hoxc11	homeobox C11	78.95	9.01E-05
Gtlf3b	Protein Gtlf3b	18.66	9.15E-04
Hoxa11	Protein Hoxa10; RCG52455	12.66	2.69E-04
H2AFJ	H2A.J histone	11.65	3.39E-05
Actc1	Actin, alpha cardiac muscle 1	11.12	4.81E-02
Hoxa11	Protein Hoxa10; RCG52455	10.61	6.65E-05
Krt5	keratin, type II cytoskeletal 5	10.56	4.39E-02
HBB	Hemoglobin subunit beta-2	10.06	6.64E-03
Hoxa11	homeobox A11	7.93	2.07E-03
Cd52	CAMPATH-1 antigen precursor	6.81	8.26E-03
Hoxd10	homeo box D10	6.78	4.48E-03

Fam167b	family with sequence similarity 167, member B	6.70	2.29E-02
Hbz	hemoglobin, zeta	6.66	2.43E-02
Hoxc10	Homeo box C10	5.14	3.69E-03
Alas2	5-aminolevulinate synthase, erythroid-specific, mitochondrial	5.12	6.27E-03
Hba-a2	Hemoglobin subunit alpha-1/2	5.07	5.27E-03
Hbe1	hemoglobin, epsilon 1	5.07	3.17E-02
Slc4a1	Band 3 anion transport protein	4.85	8.24E-03
Hba2	Hemoglobin subunit alpha-1/2	4.73	5.59E-03
Polr2l	RNA Polymerase II, I And III Subunit L	4.69	1.09E-02
Hbb-b1	hemoglobin, beta adult major chain	4.67	3.20E-03
Clip3	Protein Clip3	4.64	6.93E-02
HBA1	globin, alpha	4.60	5.20E-03
Lgals5	galectin-5	4.47	9.25E-04
Ptgds	Prostaglandin-H2 D-isomerase	-86.34	9.95E-06
Eno2	Enolase 2	-11.07	5.93E-04
Rps20	40S ribosomal protein S20	-9.70	7.81E-04
Hoxc5	homeo box C5	-7.93	1.40E-04
Lynx1	ly-6/neurotoxin-like protein 1 precursor	-7.13	3.60E-03
Calca	Calcitonin	-6.87	8.21E-04
Slc13a4	solute carrier family 13 member 4 precursor	-6.74	8.64E-05
Gm10068	predicted gene 10068	-6.63	3.31E-03
Slc22a6	Solute carrier family 22 member 6	-6.07	1.02E-04
Gng4	Guanine nucleotide-binding protein subunit gamma	-5.46	3.17E-03
Hoxc8	homeobox protein Hox-C8	-4.89	4.74E-05
Pla2g3	group 3 secretory phospholipase A2 precursor	-4.79	4.52E-04
Defb10	Beta-defensin 10	-4.71	2.29E-03
Nefh	neurofilament heavy polypeptide	-4.69	7.48E-04
Slc6a13	Sodium- and chloride-dependent GABA transporter 2	-4.68	5.09E-05
Hoxc4	homeobox protein Hox-C4	-4.55	1.23E-04
Slc16a11	solute carrier family 16, member 11	-4.49	5.94E-04
Reg3b	Regenerating islet-derived protein 3-beta	-4.30	1.56E-02
Tnc	tenascin C precursor	-4.14	1.97E-03
Kcnk9	Potassium channel subfamily K member 9	-4.04	2.45E-03
Hoxc6	Homeo box C6	-4.00	5.30E-04
Gjb6	gap junction beta-6 protein	-3.99	4.03E-03
Arhgap36	Rho GTPase Activating Protein 36	-3.96	3.49E-02
NUPR1	Nuclear protein 1	-3.86	3.15E-03
Syt2	synaptotagmin-2	-3.80	2.57E-03

Table S3. Top 25 upregulated and downregulated genes between MMC and control groups at E20.

Gene Symbol	Full Gene Name	Fold Change	P value
Hoxc11	homeobox C11	54.19	2.59E-03
Krt5	keratin, type II cytoskeletal 5	51.42	2.67E-02
Krt14	Keratin, type I cytoskeletal 14	22.17	3.03E-02
Polr2l	RNA Polymerase II, I And III Subunit L	15.90	3.36E-02
Hoxc12	homeobox protein Hox-C12	13.79	3.20E-02
Hoxa11	homeobox protein Hox a10	9.33	7.79E-03
Hoxa11	homeobox A11	8.91	3.33E-03
Hoxa11	homeobox protien Hox a10	7.67	3.30E-03
Lgals3	Galectin-3	7.64	7.48E-03
RT1-Da	RT1 class II, locus Da precursor	6.68	2.48E-03
Lyz2	lysozyme C-1 precursor	6.07	4.74E-02
Plaur	urokinase plasminogen activator surface receptor isoform 2 precursor	5.90	3.35E-02
Fcnb	Ficolin-2	5.55	2.33E-02
Prdm12	PR domain zinc finger protein 12	5.14	7.06E-03
RPS18	40S ribosomal protein S18	4.92	3.93E-02
FZD8	Frizzled 8	4.84	7.16E-03
RGD1559482	immune activating receptor CD300d1 precursor	4.70	2.06E-02
Ntrk1	High affinity nerve growth factor receptor	4.63	5.14E-03
Cd74	H-2 class II histocompatibility antigen gamma chain	4.61	2.53E-03
Hoxd8	homeobox D8	4.59	2.50E-02
Ccl12	chemokine (C-C motif) ligand 12 precursor	4.52	1.90E-02
Hist1h2ail	Histone H3.1	4.42	6.48E-04
Hspb1	heat shock protein beta-1	4.29	2.55E-02
Plau	urokinase-type plasminogen activator precursor	4.24	2.53E-02
Tlx2	T-cell leukemia, homeobox 2	4.21	3.02E-02
Pla2g3	group 3 secretory phospholipase A2 precursor	-12.18	8.95E-03
Hoxb5	homeo box B5	-10.21	2.15E-02
Hoxc4	homeobox protein Hox-C4	-9.16	1.32E-03
Mobp	Myelin-associated oligodendrocyte basic protein	-8.34	1.89E-04
Nkx6-2	NK6 homeobox 2	-7.60	1.96E-03
Slc13a4	solute carrier family 13 member 4 precursor	-7.59	1.18E-02

Slc22a6	Solute carrier family 22 member 6	-6.89	5.63E-04
Slc6a13	Sodium- and chloride-dependent GABA transporter 2	-6.83	1.30E-03
Trh	Prothyroliberin Thyroliberin	-6.44	1.74E-02
Mbp	Myelin basic protein S	-6.42	4.62E-03
Gpr17	Uracil nucleotide/cysteinyl leukotriene receptor	-6.30	3.57E-02
Bcas1	breast carcinoma-amplified sequence 1 homolog	-6.03	2.88E-02
Prok1	Prokineticin-1	-5.70	8.07E-03
Crygn	gamma-crystallin N	-5.45	2.89E-02
Pnlip	pancreatic triacylglycerol lipase precursor	-5.43	1.72E-03
Hoxc5	homeo box C5	-5.37	1.09E-03
Nkx6-3	homeobox protein Nkx-6.3	-5.34	9.69E-03
Tnks2	Protein Tnks2	-5.31	1.48E-03
Hoxa2	homeobox protein Hox-A2	-4.94	2.14E-02
Bmp6	Bone morphogenetic protein 6	-4.92	3.10E-03
Hoxa3	homeobox protein Hox-A3	-4.83	1.36E-02
Plp1	Myelin proteolipid protein	-4.76	4.45E-03
Dbh	dopamine beta-hydroxylase	-4.43	1.53E-02
Tmco3	Transmembrane And Coiled-Coil Domains 3	-4.29	1.09E-02
Lin28b	Lin-28 Homolog B	-4.27	4.19E-02

Table S4. Top 25 upregulated and downregulated genes between control and vehicle groups at E15.

Gene Symbol	Full Gene Name	Fold Change	P value
RPS18	40S ribosomal protein S18	5.57	2.23E-02
Actc1	Actin, alpha cardiac muscle 1	5.35	5.50E-03
Rps24	40S ribosomal protein S24	3.32	2.63E-03
RGD1563528	brain protein 44-like protein 2	3.20	3.76E-04
Tnnc2	troponin C, skeletal muscle	3.19	3.23E-02
Lrrc23	Leucine rich repeat containing 23	3.18	1.81E-03
Riiad1	RIIa domain-containing protein	2.87	3.56E-04
ND5	NADH dehydrogenase subunit 5 (mitochondrion)	2.79	4.91E-02
Trav10d	T cell receptor alpha variable 10D	2.61	2.04E-03
Tsga14	Testis Specific Protein A14	2.61	3.88E-02
PEX12	Peroxisomal biogenesis factor 12	2.61	3.88E-02
ATP8	ATP synthase F0 subunit 8 (mitochondrion)	2.60	1.09E-02
Hist3h2bb	histone cluster 3, H2bb	2.55	1.72E-02
Myog	myogenin	2.53	5.22E-02
Mt2A	Metallothionein-2	2.52	4.33E-02
ATP5F1E	ATP synthase subunit epsilon, mitochondrial	2.35	6.78E-04
RPS29	40S ribosomal protein S29	2.29	2.79E-02
Rnase12	Ribonuclease-like protein 12	2.24	2.38E-02
ND6	NADH dehydrogenase subunit 6 (mitochondrion)	2.22	1.21E-01
Rps20	40S ribosomal protein S20	2.21	3.35E-02
Apoc3	Apolipoprotein C-III	2.19	3.42E-03
Cd52	CAMPATH-1 antigen precursor	2.15	3.85E-02
Rpl32-ps1	Ribosomal protein L32, psueodgene 1	2.12	4.31E-02
Pip5kl1	phosphatidylinositol-4-phosphate 5-kinase-like 1	2.02	4.05E-03
Cited4	Cbp/p300-interacting transactivator 4	2.01	1.28E-02
EF1A	Elongation factor 1-alpha	-213.11	1.28E-05
Hoxc10	Homeo box C10	-46.52	1.23E-03
Hoxd10	homeo box D10	-33.73	1.35E-06
Hoxc12	homeobox protein Hox-C12	-28.62	1.30E-05
Hoxd9	Homeobox protein Hox-D9	-24.44	1.24E-03
Hoxc11	homeobox C11	-23.20	1.10E-06
Hoxa11	Homeo box A10	-4.61	2.61E-03

Colq	acetylcholinesterase collagenic tail peptide precursor	-2.99	2.67E-03
MAP2K3	Mitogen activated protein kinase 3	-2.77	1.29E-02
Hoxa11	homeobox A11	-2.66	8.22E-03
Hist2h2ac	Histone H2A	-2.34	2.20E-02
Ptpro	receptor-type tyrosine-protein phosphatase O precursor	-2.34	1.74E-04
Pmaip1	Phorbol-12-myristate-13-acetate-induced protein 1	-2.33	9.94E-04
Rpl32	ribosomal protein L32	-2.30	2.38E-03
HMGB3	High mobility group protein B3	-2.26	5.78E-02
Wfdc10	WAP four-disulfide core domain 10 precursor	-2.23	8.54E-03
Capg	Macrophage-capping protein	-2.20	1.04E-03
Ifitm1	interferon-induced transmembrane protein 1	-2.13	1.99E-03
Retsat	all-trans-retinol 13,14-reductase precursor	-2.12	4.60E-04
Hoxa9	Homeo box A9	-2.12	1.86E-03
Hist1h2bl	histone H2B type 1	-2.09	5.72E-03
Ptma	Prothymosin alpha Thymosin alpha	-2.06	3.74E-02
Slc25a22	Solute carrier family 25 (Mitochondrial carrier, glutamate), member 22	-2.04	3.61E-03
Pax7	paired box protein Pax-7	-2.02	1.10E-03
Tmem200b	transmembrane protein 200B	-1.98	1.07E-02

Table S5. Top 25 upregulated and downregulated genes between vehicle and control groups at E17.

Gene Symbol	Full Gene Name	Fold Change	P value
Myl2	myosin regulatory light chain 2, ventricular/cardiac muscle isoform	6.51	3.71E-03
Retsat	all-trans-retinol 13,14-reductase precursor	4.47	1.66E-05
Capg	Macrophage-capping protein	3.49	6.72E-05
Defb11	Beta-defensin 11	2.90	3.44E-03
Trav3-4	T cell receptor alpha variable 3-4	2.64	9.00E-05
Rgs13	Regulator Of G Protein Signaling 13	2.43	1.62E-02
HIAT1	Hippocampus abundant gene transcript 1	2.23	2.67E-02
PORF1	Putative preoptic regulatory factor 1	2.18	2.16E-04
Pde6g	phosphodiesterase 6G, cGMP-specific, rod, gamma	2.09	1.42E-02
Fosl2	fos-related antigen 2 isoform 1	2.07	1.80E-02
Diap2	diaphanous homolog 2 (Drosophila)]	2.00	3.87E-02
Rps24	40S ribosomal protein S24	1.98	2.73E-02
Scgb1c1	Secretoglobin family 1C member 1	1.90	1.42E-03
Tm4sf19	transmembrane 4 L6 family member 19	1.86	9.06E-03
Fam70b	Family With Sequence Similarity 70, Member B	1.85	1.27E-02
Relb	avian reticuloendotheliosis viral (v-rel) oncogene related B	1.82	4.94E-03
Ifitm3	Interferon-induced transmembrane protein 3	1.82	1.40E-02
Thbd	thrombomodulin precursor	1.81	1.04E-02
Immp2l	Inner Mitochondrial Membrane Peptidase Subunit 2	1.80	4.49E-02
Il18	Interleukin-18	1.75	3.60E-02
Foxa1	hepatocyte nuclear factor 3-alpha	1.75	1.81E-02
Fabp4	fatty acid-binding protein, adipocyte	1.74	4.12E-03
Itih3	Inter-alpha-trypsin inhibitor heavy chain H3	1.73	2.38E-02
Slc22a8	Solute carrier family 22 member 8	1.70	1.40E-02
Fam64a	Family With Sequence Similarity 64 Member A	1.69	5.52E-03
Hoxc12	homeobox protein Hox-C12	-22.90	2.69E-03
Gtlf3b	gene trap locus F3b	-10.78	3.43E-05
Hoxc11	homeobox C11	-10.22	1.98E-03
SDHAF1	Succinate dehydrogenase assembly factor 1, mitochondrial	-6.07	3.09E-02
S100a8	Protein S100-A8	-4.00	1.33E-02

Cd52	CAMPATH-1 antigen precursor	-3.34	3.04E-02
RPS29	40S ribosomal protein S29	-3.23	2.60E-03
Hoxa11	homeobox A11	-3.16	7.59E-04
Hbb	Hemoglobin subunit beta-1	-2.92	5.27E-03
Hbb-b1	hemoglobin, beta adult major chain	-2.82	3.63E-03
Hba-a2	Hemoglobin subunit alpha-1/2	-2.80	1.66E-02
Alas2	5-aminolevulinate synthase, erythroid-specific, mitochondrial	-2.75	2.94E-02
Hba1	globin, alpha	-2.65	2.81E-02
Hba2	Hemoglobin subunit alpha-1/2	-2.65	1.88E-02
FZD8	Frizzled 8	-2.62	3.25E-03
Lgals5	galectin-5	-2.54	2.42E-03
MT1	Metallothionein-1	-2.49	2.59E-03
Ahsp	alpha-hemoglobin-stabilizing protein	-2.48	9.60E-03
Hoxd10	homeo box D10	-2.40	4.56E-02
Fam46c	family with sequence similarity 46, member C	-2.33	2.10E-02
Hbb-b1	Hemoglobin subunit beta-1	-2.32	6.19E-03
Hbb-b2	Hemoglobin subunit beta-2	-2.28	7.83E-03
Rhd	Blood group Rh(D) polypeptide	-2.24	4.81E-02
Lsm6	Protein Lsm6	-2.24	4.72E-03
Wfdc10	WAP four-disulfide core domain 10 precursor	-2.15	8.20E-03

Table S6. Top 25 upregulated and downregulated genes between vehicle and control groups at E20.

Gene Symbol	Full Gene Name	Fold Change	P value
EF1A	Elongation factor 1-alpha	104.82	1.65E-04
Prok1	Prokineticin-1	5.80	1.56E-02
TMSB10	Thymosin Beta-10	4.76	3.99E-04
Immp2l	Inner mitochondrial membrane protease subunit 2	4.58	8.32E-03
Hoxc5	homeo box C5	3.58	4.50E-02
Agbl3	Protein Agbl3	3.52	5.00E-03
Prok2	Prokineticin-2	3.50	3.54E-04
Hdac10	Histone deacetylase 10	3.26	3.41E-02
Mepce	Methylphosphate capping enzyme	3.23	4.59E-04
Fam227a	Family with sequence similarity 227 member A	3.23	3.48E-02
Slc2a3	solute carrier family 2, facilitated glucose transporter member 3	3.03	1.13E-03
RPL27	60S ribosomal protein L27	2.97	3.04E-02
Plekhn1	pleckstrin homology domain containing, family N member 1	2.96	3.69E-02
ZFP692	zinc finger protein 692	2.93	3.69E-02
Irf3	interferon regulatory factor 3	2.87	7.27E-03
SNIP1	Smad nuclear interacting protein 1	2.83	3.03E-03
Pdzd7	PDZ domain containing 7	2.74	4.67E-02
Spag4	sperm-associated antigen 4 protein	2.70	9.00E-03
Crygd	Gamma-crystallin D	2.62	2.35E-02
Mrgbp	MRG domain binding protein	2.62	2.33E-02
Pdia2	protein disulfide-isomerase A2 precursor	2.61	3.25E-02
Ccdc142	coiled coil domain containing 142	2.60	2.94E-02
Lgals5	galectin-5	2.59	4.82E-02
Leng8	leukocyte receptor cluster member 8	2.57	4.72E-02
Wsb1	WD repeat and SOCS box-containing 1 isoform 1	2.56	3.70E-02
Mustn1	Musculoskeletal embryonic nuclear protein 1	-11.68	5.55E-03
NCBP2	Nuclear cap binding protein subunit 2	-11.21	2.95E-03
Ly6g6d	Lymphocyte antigen 6 complex locus protein G6d	-3.83	1.43E-02
Hist1h2ai	histone cluster 1, H2an	-3.39	4.49E-04
Six2	homeobox protein SIX2	-2.84	1.98E-03
Tsga14	Testis Specific Protein A14	-2.71	7.66E-03
Hist1h2ail1	Histone H3.1	-2.66	5.79E-03

Pnpla2	Patatin-like phospholipase domain-containing protein 2	-2.60	1.98E-02
RPS29	40S ribosomal protein S29	-2.43	3.27E-02
En1	Engrailed homeobox 1	-2.38	2.06E-02
Plekhf1	Pleckstrin homology domain-containing family F member 1	-2.32	1.17E-02
Fgf7	fibroblast growth factor 7 precursor	-2.19	2.56E-02
Fst	Follistatin	-2.19	9.79E-03
Fosl2	fos-related antigen 2 isoform 1	-2.13	1.03E-02
Nes	nestin	-2.10	9.47E-03
Il21	Interleukin-21	-2.08	2.85E-02
Pdgfrl	Platelet-derived growth factor receptor-like protein	-2.05	4.58E-02
Mt4	metallothionein-4	-2.03	4.09E-03
Rhobtb3	rho-related BTB domain-containing protein 3	-2.01	2.54E-02
Nav1	neuron navigator 1	-2.01	2.66E-02
Rps20	40S ribosomal protein S20	-2.00	1.89E-03
Hist1h2bd	histone cluster 1, H2bd	-1.97	4.91E-02
S100a4	Protein S100-A4	-1.93	2.44E-02
Tal2	T-cell acute lymphocytic leukemia 2	-1.91	5.61E-03
Ogn	mimecan precursor	-1.87	1.36E-02
